# Carbon nanofiber/taurine-catalyzed synthesis of coumarin and 1,2,4,5-tetra-substituted imidazole derivatives under metal-free conditions

**DOI:** 10.1038/s41598-024-61249-2

**Published:** 2024-05-09

**Authors:** Dina Mallah, Bi Bi Fatemeh Mirjalili, Abdolhamid Bamoniri

**Affiliations:** 1 Department of Chemistry, College of Science, Yazd University, P.O. Box 89195-741, Yazd, Islamic Republic of Iran; 2 Department of Organic Chemistry, Faculty of Chemistry, University of Kashan, Kashan, Islamic Republic of Iran

**Keywords:** Chemistry, Catalysis, Catalyst synthesis

## Abstract

The main subject of this research is the development of a suitable, efficient, and biocompatible carbon nanofiber-based catalytic system for the synthesis of coumarin and 1,2,4,5-tetra-substituted imidazoles. Brønsted acid carbon nanofiber/taurine catalyst was made during three steps: acid treatment, acylation, and then amination. The basic principles and general advantages of the synthesis method are elaborated. The acidity of the prepared nano-catalyst was investigated using the Hammet acidity technique and UV–Vis spectroscopy, and the *H*_0_ value for 5 × 10^–2^ mg/mL of CNF/T in 0.3 mM 4-nitroaniline solution was determined to be 1.47. The structure of the catalyst was successfully characterized using FT-IR, TGA, FESEM, XRD, TEM, EDX, EDS-MAP, BET, and XPS techniques. Here, we report the ability of carbon nanofiber/taurine as a Brønsted acid catalyst for the synthesis of coumarins and 1,2,4,5-tetra-substituted imidazole through a metal-free, cost-effective, and biocompatible multicomponent route. Among the advantages of this protocol are reaction time, excellent efficiency, reusability, and high activity of the catalyst.

## Introduction

Carbon nanofibers have attracted much attention recently due to their unique characteristics^1,2^. In particular, the inertness of the surface, the presence of stabilizing surface groups, and conductivity properties have been reported according to the heterogeneous catalyst^3^. Hence, the modification of carbon-based materials, especially carbon nanofibers, and their use for catalysts, is an interesting development in recent decades^4^. Carbon nanofibers have high surface porosity and high surface area. Due to the inertness of the surface, the carbon surface avoids unnecessary chemical reactions with the reactant, therefore, it is a suitable support for catalysis, which can be a suitable and cheap alternative to the support of conventional catalysts, because carbon nanofibers are produced from biomass^5–7^.

Taurine as an amino sulfonic acid is essential for proper heart function, healthy sleep, and promoting calmness. Taurine is found in large amounts in the brain, retina heart, and blood cells called platelets. Several reports have suggested that taurine, in addition to its medicinal properties, also plays a role in the preparation of catalysts and the synthesis of heterocyclic compounds^8^.

Organic chemists have shown extensive attention to heterocycles due to their wide applications and diverse biological properties^9^. Among known heterocyclic compounds, coumarin and imidazoles are of great importance^10,11^. Coumarin is a natural compound and a group of compounds called benzopyrone, which was first synthesized in 1868 by Perkin^12^. Coumarin derivatives have many applications including anti-tumor, anti-HIV, anti-bacterial, and anti-inflammatory, dyes, and are also known as fat-reducing agents^13–18^. Several methods have been reported for the synthesis of coumarins, including the Pechmann, Wittig, Perkin reaction, Knoevenagel reaction, Kostanecki-Robinson, and Reformatsky reactions^16^. Coumarins are known by different names, including 2*H*-1-benzopyran-2-one, 1,2-benzopyrone, cis-*o*-coumarinicacidlactone, coumarinicanhydride, *o*-hydroxycinnamicacidlactone, and 2-oxo-1-benzopyrones^13–16^. These compounds consist of fused rings of benzene and α-pyrone and belong to a class of flavonoids and a type of benzo-2-pyrone. In Pechmann condensation for the synthesis of coumarin, phenols, and *β*-ketoesters or *α*,*β*-unsaturated carboxylic acids are often used. Previously, various acid catalysts such as γ-Fe_2_O_3_@HAp-Ag NPs^19^, PMA/Cr–Mg-MOF^20^, Zn_0.925_Ti_0.075_O^21^, [Et_3_NH][HSO_4_]^22^, ChCl.2SnCl_2_^23^, (ZrO_2_-TiO_2_, ZrO_2_-ZnO_,_ and ZrO_2_/cellulose)^24^, MNESA^25^, and CNC‐ MPD‐Pd^26^ were used for the synthesis of coumarins.

Biologically active compounds mainly have a polycyclic heteroatom structure containing N^10,11^. Among the 5-membered heteroatom ring structures with N, the imidazole nucleus has attracted the attention of chemists due to the high therapeutic properties of imidazole-containing drugs in the medical field. In the field of medicine, the properties of imidazoles include anti-cancer, *β*-lactamase inhibitors, anti-aging agents, heme oxygenase inhibitors, antibacterial, anti-inflammatory, anti-diabetes, anti-tuberculosis, and malaria^10,27^. Imidazole was first synthesized in 1858 by Heinrich Debus using diketone, formaldehyde, and ammonia. Various methods for the synthesis of multi-functionalized imidazole derivatives have been reported, including Van Leusen synthesis, Wallach synthesis, Marckwald synthesis, and Debus-Radziszewski synthesis^28^. Imidazole derivatives have been synthesized in the presence of various acidic and basic catalysts including ZSM-11 zeolite^29^, [Bmim]HSO_4_^30^, Zn(OAc)_2_.2H_2_O^31^, 1,4- dimethylpiperaziniumdihydrosulfate^32^, [2,6-DMPyH]C(NO_2_)_3_^33^, Cu_0.9_Fe_0.1_@RCAC^34^, RHCAC^35^, pyridinium hydrogen sulfate^36^, and CTSA^37^.

**Figure 1:**
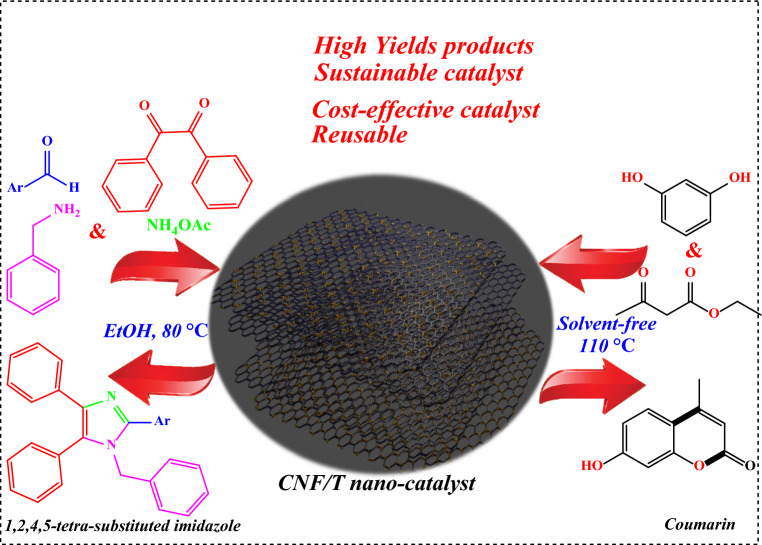
Application of CNF/T nano-catalyst.

In the present research work, we have prepared for the first time, a Brønsted acidic carbon nanofiber functionalized taurine named carbon nanofiber/taurine (CNF/T) for the synthesis of coumarin through Pechmann condensation under mild reaction conditions. In the following work, we report the CNF/T catalyst for the synthesis of 1,2,4,5-tetra-substituted imidazole (Fig. 1.). The structure of the prepared CNF/T nano-catalyst was confirmed using FT-IR, TGA, FESEM, XRD, TEM, EDX, EDS-MAP, BET, and XPS techniques.

## Results and discussions

**Figure 2:**
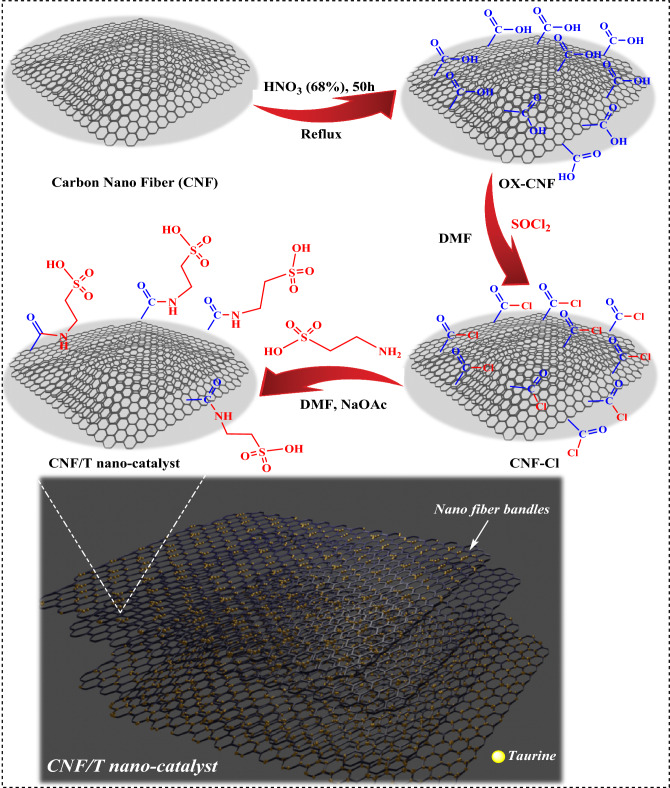
Stepwise preparation of CNF/T nano-catalyst.

The CNF/T was prepared (Fig. 2) and identified using different techniques such as FT-IR, TGA, FESEM, XRD, TEM, EDX, EDS-MAP, BET, and XPS.

### FT-IR analysis of CNF/T nano-catalyst

**Figure 3:**
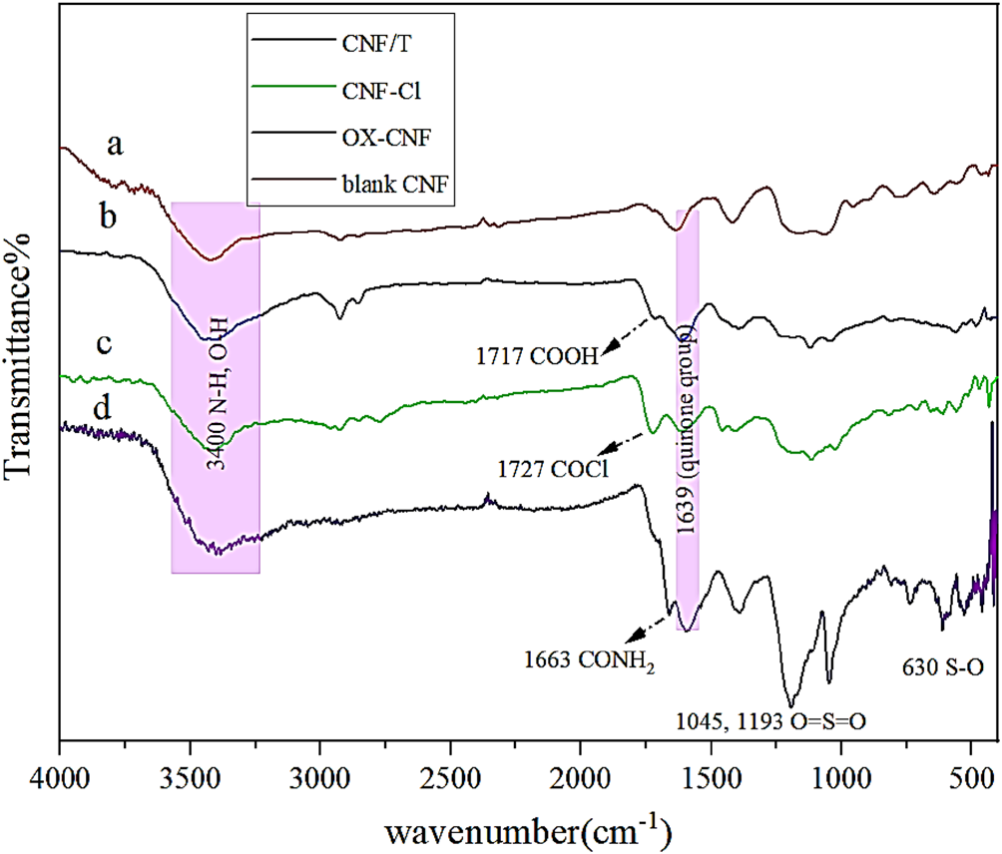
FT-IR spectra of (**a**) blank CNF, (**b**) OX-CNF, (**c**) CNF-Cl, and (**d**) CNF/T.

FT-IR spectrum can easily confirm and evaluate the step-by-step fabrication of CNF/T nano-catalyst. For this purpose, the spectrum of CNF/T was compared with the spectra of blank CNF, acid-treated CNF, and acylated CNF (Fig. 3). As can be seen, all spectra show a peak at 1639 cm^−1^ and 3400 cm^−1^ corresponding to the C=O stretching vibration of quinone groups and the hydroxyl group (O–H) stretching vibration, respectively. When nanocarbon fibers are treated with acid and oxidized, a band appears at 1717 cm^−1^, which is related to the C=O stretching vibration mode of the carboxylic acid group (Fig. 3b). In the chlorination step of CNF (CNF-Cl), the distinct peak located at 1717 cm^−1^ corresponding to C=O carboxylic acid in Fig. 3b, shifted to 1727 cm^−1^, which can confirm the formation of the COCl functional group (Fig. 3c). In Fig. 3d, the presence of a specific peak at 1663 cm^−1^ is related to the stretching vibration of the CONH_2_ group, the presence of a band at 630 cm^−1^ is related to the stretching vibration of S–O, as the symmetric and asymmetric stretching bands of O=S=O at 1045 cm^−1^ and 1193 cm^−1^, respectively, can be evidence for this claim that the CNF/T nano-catalyst is prepared correctly.

### TGA of CNF/T

**Figure 4:**
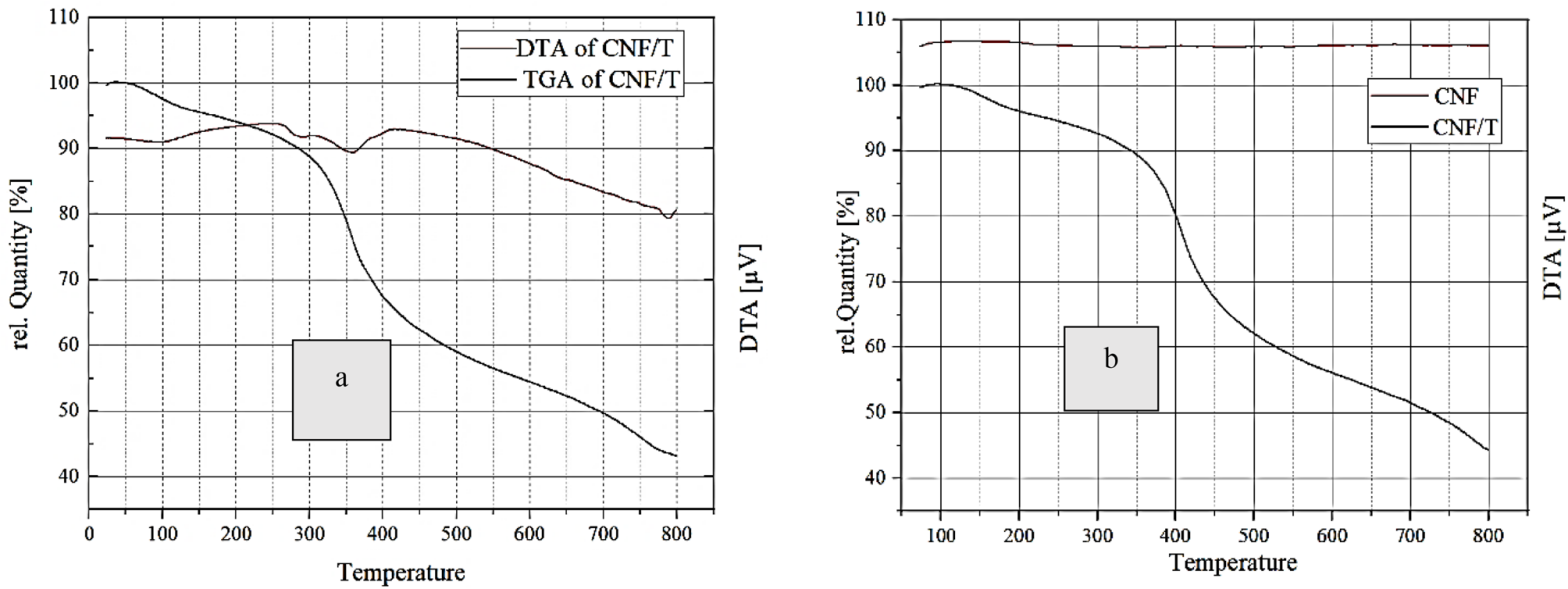
(**a**) TGA/DTA curves of CNF/T nano-catalyst, (**b**) TGA curves of pure CNF and CNF/T.

In Fig. 4, the thermogravimetric behavior of CNF/T nano-catalyst is defined as a result of thermal decomposition in the range of 20–800 °C. TGA curves were measured under N_2_ flow in the temperature range of 20–800 °C for CNF and CNF/T nano-catalyst. As can be seen, there are two main stages of weight loss. The first one at a temperature less than 100 °C is related to the loss of residual solvent and the remaining two cases are related to the decomposition of the organic group (150–400 °C) and the carbon nanofiber (450–700 °C) respectively (Fig. 4a). Figure 4b shows the thermogravimetric results of carbon nanofiber and CNF/T. As can be seen, CNF degradation in CNF/T starts at about 430°C while pure CNF starts to lose mass at 390 °C^38^ which shows that functionalized CNF has higher thermal stability compared to pure CNF. The different thermal behavior of these two samples is a confirmation of the successful completion of this modification.

### FESEM and TEM of CNF/T nano-catalyst

**Figure 5:**
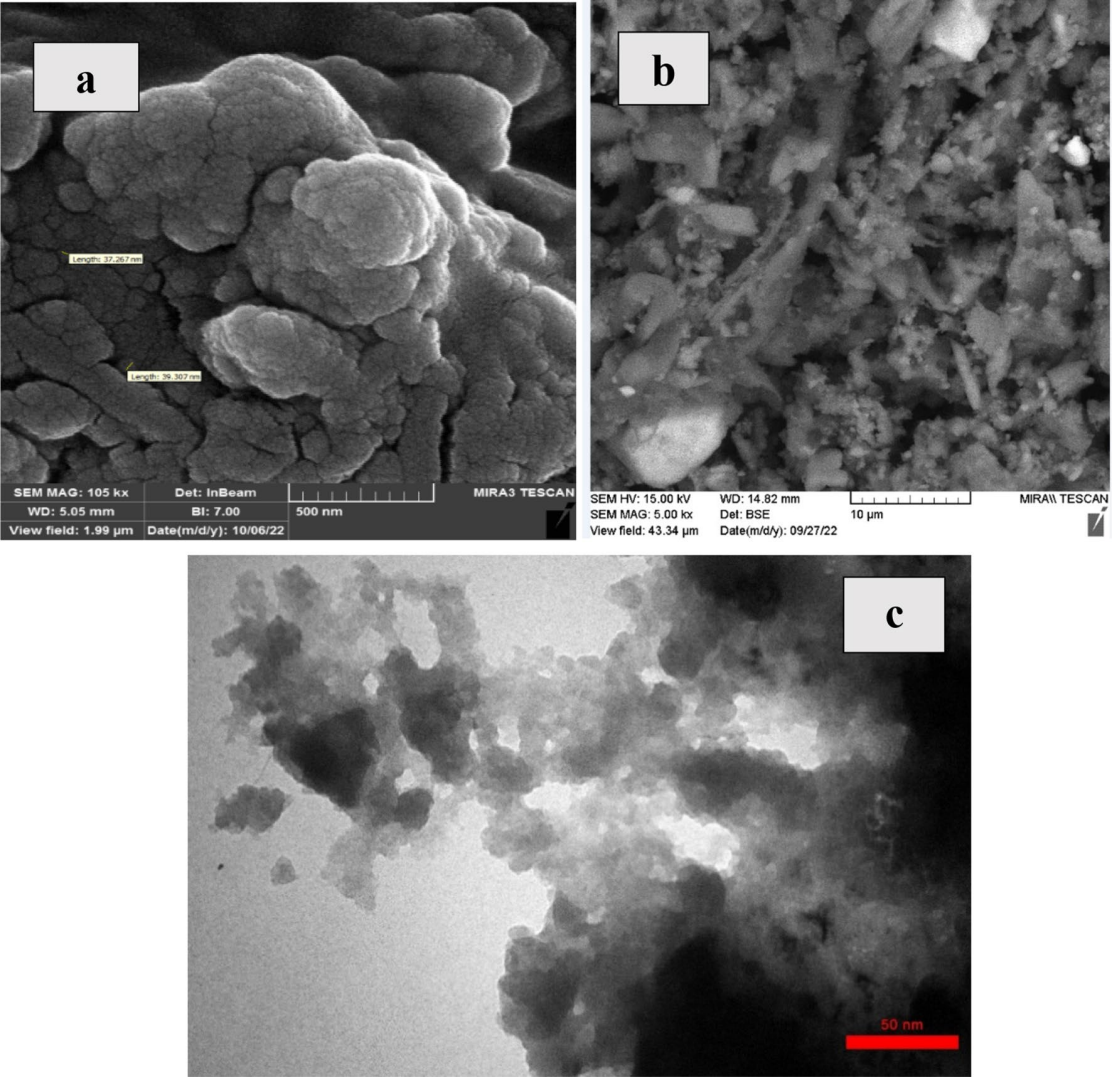
(**a**) FESEM of CNF/T nano-catalyst at high magnification, (**b**) SEM-Image captured at low magnification (**c**) TEM of CNF/T.

To show the morphology of the CNF/T nano-catalyst, FESEM was performed. As shown in Fig. 5a,b, CNF/T is a nanoparticle with a diameter in the range of 37–39 nm. Meanwhile, The TEM image of CNF/T shows that this catalyst is a nanoparticle (Fig. 5c).

### PXRD (powder X-ray diffraction) of CNF/T

**Figure 6:**
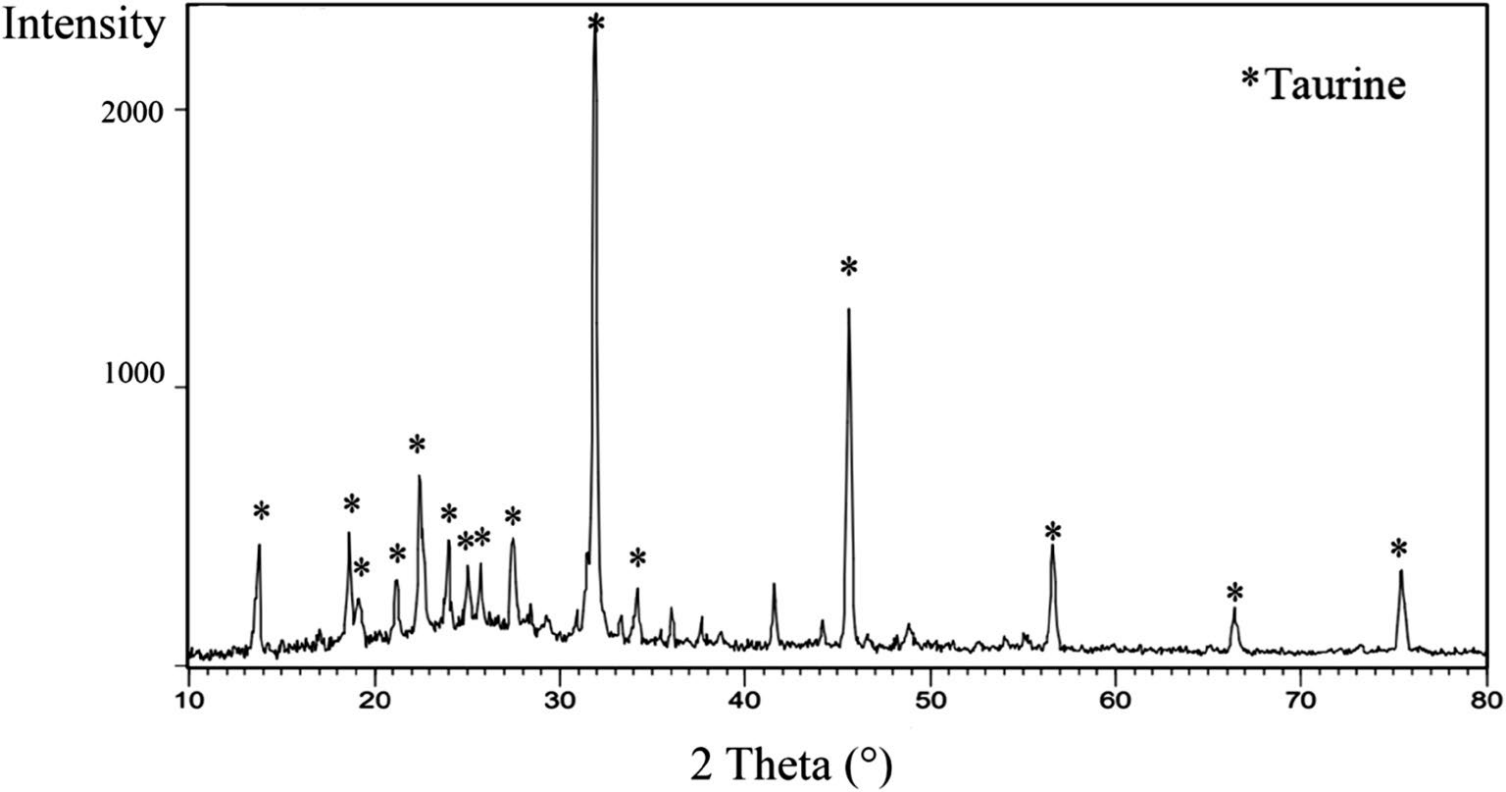
PXRD pattern of CNF/T nano-catalyst.

The crystal structure of the CNF/T nano-catalyst was determined using the X-ray diffraction method (Fig. 6). As shown, carbon nanofibers show an amorphous structure and there is a peak in the range of 25° without sharp diffraction peaks which is consistent with the reported XRD spectrum of carbon nanofibers^39^. After the functionalization of taurine on CNF, the CNF/T nano-catalyst was successfully prepared. The prepared nano-catalyst shows similar diffraction peaks compared to taurine and CNF^40,41^.

### EDX and EDS-map of CNF/T

**Figure 7:**
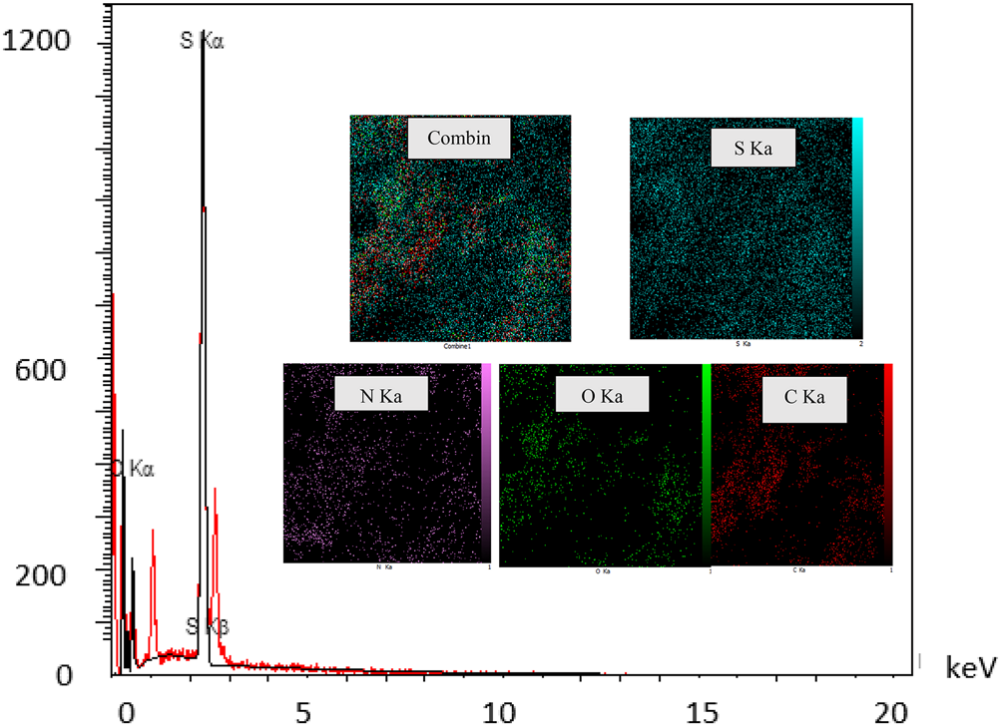
Electron dispersed X-ray (EDX) analysis and maps (MAG: 5.00 kx) of CNF/T nano-catalyst.

EDX analysis and elemental mapping of CNF/T were used to determine the positions of elemental composition and elemental percentage composition (Fig. 7). The obtained results confirm the presence of elements C, N, O, and S in the prepared catalyst with 44.68, 21.9, 17.14, and 16.28%. Figure 7 shows the SEM elemental mapping images for the functionalized CNF nano-catalyst with taurine. Maps of Carbon (C), Nitrogen (N), Oxygen (O), and Sulfur (S) show a uniform distribution of elements. According to Fig. 7, it can be concluded that taurine is immobilized on the carbon nanofiber surface.

### BET of CNF/T

**Figure 8:**
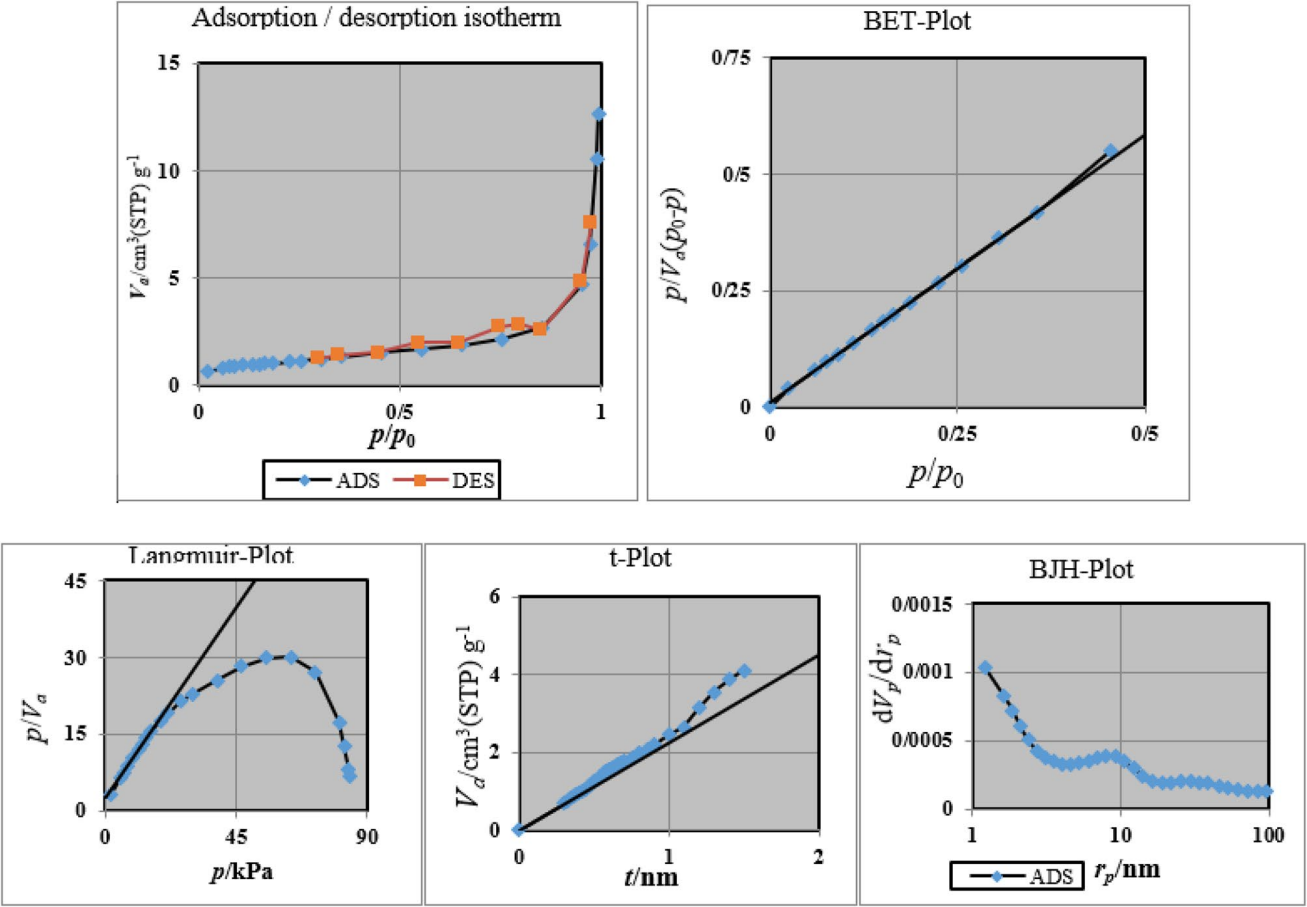
N_2_ adsorption (blue line)–desorption (red line) isotherm and corresponding diagrams pore size distributions (BJH, BET, Langmuir, *t*-plot).

Nitrogen adsorption at 77 K was performed to evaluate the porosity development and confirm the CNF/T mesoporous structure, and the results are depicted in Fig. 8 with the parameters summarized in Table 1. CNF/T nano-catalyst shows a typical isotherm of IV type (Fig. 8) according to the IUPAC classification, which is characteristic of mesoporous materials, the diameter of the pore is 18.559 nm. The textural properties of the studied CNF/T such as surface area, mean pore diameter, total pore volume, and BJH are shown in Table 1, which are reported in 3.76 m^2^ g^−1^, 18.559 nm, 0.017445 cm^3^ g^−1^, 0.017134 cm^3^ g^−1^, respectively.

**Table 1:** Parameters obtained from porosity analysis.

BET	
*V*_*m*_	0.8638 [cm^3^(STP) g^−1^]
a_s, BET_	3.7598 [m^2^ g^−1^]
*C*	112.55
Total pore volume (*p*/*p*_0_ = 0.990)	0.017445 [cm^3^ g^−1^]
Mean pore diameter	18.559 [nm]
Langmuir plot	
V_m_	1.2176 [cm^3^(STP) g^−1^]
a_s,Lang_	5.2997 [m^2^ g^−1^]
B	0.3382
t plot	
Plot data	Adsorption branch
a_1_	3.4748 [m^2^ g^−1^]
V_1_	0 [cm^3^ g^−1^]
BJH plot	
Plot data	Adsorption branch
V_p_	0.017134 [cm^3^ g^−1^]
*r*_*p,peak*_ (area)	1.64 [nm]
a_p_	3.5715 [m^2^ g^−1^]

### Hammet acidity function of CNF/T

**Figure 9:**
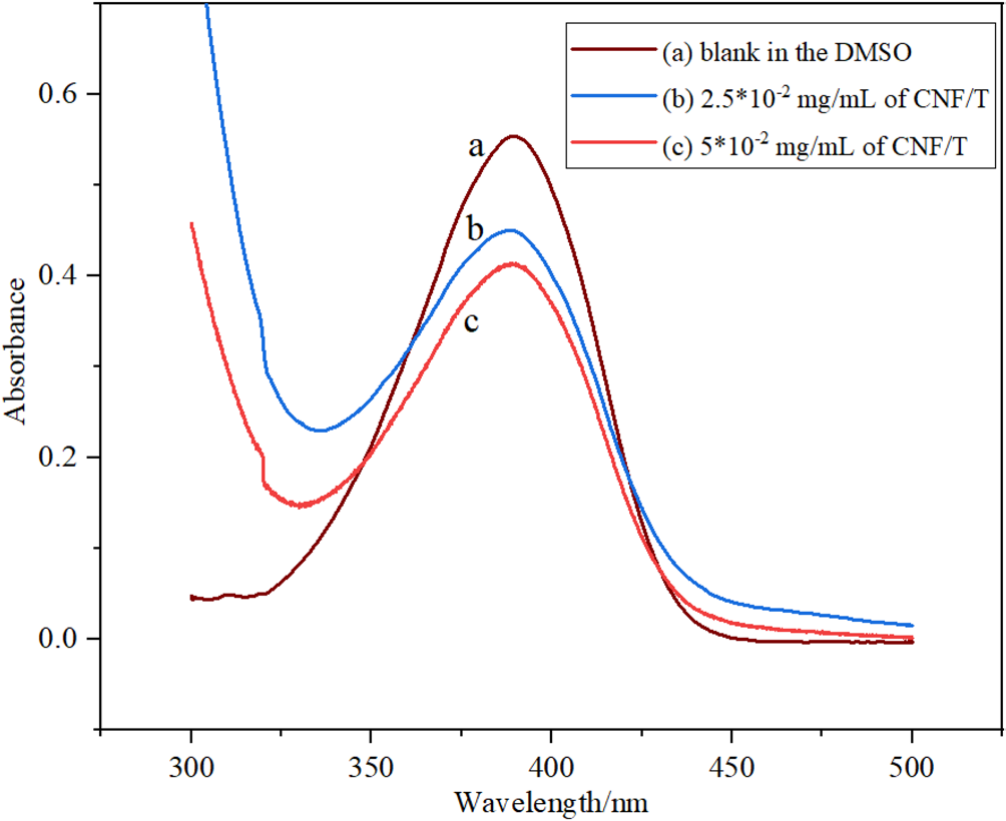
Absorption spectra of 4-nitroaniline and different amounts of CNF/T nano-catalyst.

The acidity of CNF/T was investigated using the Hammett acidity function method and UV–Vis spectroscopy, in which a base detector is used to trap the excitable proton^42^. Here, 4-nitroaniline was chosen as the indicator and DMSO was used as the solvent (Fig. 9). CNF/T nano-catalyst was dispersed in DMSO for 30 min and the resulting suspension was centrifuged to create a clear and transparent solution. The maximum absorption (*A*_max_) was observed at 0.55 at λ_max_ = 389 nm in DMSO solvent for the un-protonated form of 4-nitroaniline. The *H*_0_ values of suspension top solutions with 2.5 × 10^–2^ mg/mL and 5 × 10^–2^ mg/mL of CNF/T in 4-nitroaniline solution were determined and calculated using the ratio [In^−^]/[InH^+^] and UV–Vis spectroscopy.

This absorption decreases with the increase of CNF/T as a Brønsted acid. As is evident from Table 2 and Fig. 9, the absorption decreases with the increase of CNF/T values in the 4-nitroaniline solution. A solution of 5 × 10^–2^ mg/mL of CNF/T in 0.3 mM 4-nitroaniline with a *H*_0_ = 1.47 value was determined using Eq. (1).

1 $$ H_{0} = {\text{ pKa }} + {\text{ log}}\left( {\left[ {{\text{In}}^{ - } } \right]/\left[ {{\text{InH}}^{ + } } \right]} \right) $$

where pKa is the value of the indicator prepared in DMSO solvent and [In^−^] and [HIn^+^] are the concentration values of the protonated and un-protonated forms of the indicator in the solvent, respectively.

**Table 2:** Hammett acidity function values of various concentrations of investigated CNF/T^a^.


Entry	CNF/T (mg/mL)	*A*_max_	[In^−^] (%)	[HIn^+^] (%)	*H*_0_
1	–	0.55	100	–	–
2	2.5 × 10^–2^	0.45	81.81	18.19	1.66
3	5 × 10^–2^	0.41	74.53	25.47	1.47

### XPS (X-ray photoelectron spectroscopy) of CNF/T

**Figure 10:**
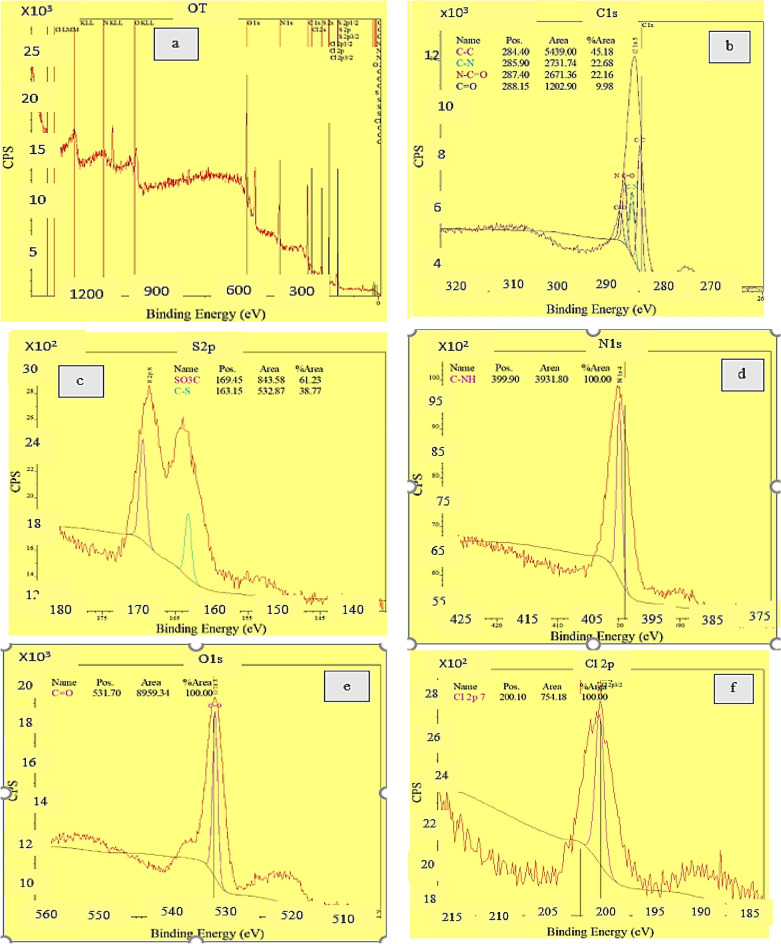
XPS spectrum of CNF/T.

XPS analysis was used to check the bond type and measure and determine the chemical composition (Fig. 10a–f). The CNF/T nano-catalyst was investigated using X-ray photoelectron spectroscopy. Examination of the CNF/T XPS spectrum shows four dominant peaks at 284 eV, 400 eV, 169 eV, 531 eV, and 200 eV, which correspond to C 1s, N 1s, S 2p, O 1s, and Cl 2p, respectively (Fig. 10a). The peak at 284 eV corresponds to C1s, which can be decomposed into four components at binding energies of 284.4 eV, 285.9 eV, 287.4 eV, and 288.15 eV, which can be attributed to bonds C–C, C–N, N–C=O, and C=O in the composition respectively (Fig. 10b). The peaks at the binding energies of 163.15 eV and 169.45 eV that appear in the S 2p spectrum for C-S and SO_3_-C, respectively (Fig. 10c). In the N 1s spectrum (Fig. 10d), the peak at 399.9 eV corresponds to the C–N bond. The peak at the binding energy of 532 eV (Fig. 10e) and 200 eV (Fig. 10f) correspond to presence of C=O and Cl in the organic compound, respectively. All these data confirm the binding of taurine to carbon nanofibers.

To identify and fully evaluate the structure of CNF/T nano-catalyst and to confirm the functionalization of treated carbon nanofiber using FT-IR, TGA, FESEM, XRD, TEM, EDX, EDS-MAP, and BET techniques, its catalytic activity for the synthesis of coumarins and 1,2,4,5-tetra-substituted imidazoles was investigated. At first, the catalytic activity of the CNF/T nano-catalyst was tested in the model reaction for the synthesis of 7-hydroxycoumarin using resorcinol and ethyl acetoacetate in the presence of CNF/T under various conditions and the results are presented in Table 3.The excellent performance of the CNF/T nano-catalyst (0.05 g) under solvent-free conditions at 110 °C during the synthesis of coumarin encouraged us to carry out their applications for a wide range of substrates including a variety of active phenols with different *β*-ketoesters. As shown in Table 3, high yields of coumarins up to 90% were obtained in the reaction of *β*-ketoesters with different phenols. Resorcinol (Table 4, entries 1–7), phloroglucinol (Table 4, entries 8–12), pyrogallol (Table 4, entry 13), and *α*-naphthol (Table 4, entry 14) reacted with a variety of *β*-ketoesters and give the corresponding coumarins with 75–90% yield. As it is evident, phenols with electron-donating substitution in the para position of the hydroxyl group (resorcinol and phloroglucinol) have higher yield and shorter time, and while *α*-naphthol has lower yield and longer time, on the other hand, spatial crowding is very effective. In such a way efficiency decreases with the increase of spatial congestion.

**Table 3:** Optimization of the reaction conditions for coumarin synthesis by the Pechmann condensation.


Entry	Conditions	Time (h)	Yield (%)^b^
	Solvent/temp. (°C)/catalyst (g)		
1	–/110/CNF/T (0.005)	12	Trace
2	–/110/CNF/T (0.01)	12	Trace
3	–/110/CNF/T (0.015)	12	Trace
4	–/110./CNF/T (0.02)	8	45
5	–/110/CNF/T (0.025)	6	56
6	–/110/CNF/T (0.03)	6	64
7	–/110/CNF/T (0.035)	5.5	68
8	–/110/CNF/T/(0.04)	5.5	74
9	–/110/CNF/T/(0.045)	4	75
**10**	–/**110/CNF/T (0.05)**	**3**	**89**
11	–/80/CNF/T (0.05)	6	38
12	–/90/CNF/T (0.05)	8	53
13	–/100/CNF/T (0.05)	3.5	65
14	EtOH/110/CNF/T (0.05)	5	65
15	EtOH: H_2_O (1:1)/110/CNF/T (0.05)	5	44
16	H_2_O/110/CNF/T (0.05)	5	32
17	–/110/–	5	Trace

Conditions: resorcinol (1 mmol), ethyl acetoacetate (1 mmol), solvent (10 ml).

Significant values are given in bold.

^a^Isolated yield.

**Table 4:** Substrate scope for the synthesis of coumarins from activated phenol and various *β*-ketoesters using 0.05 g CNF/T under solvent-free conditions.


Entry	R_1_	R_2_	R_3_	Time (h)	Yield (%)^a^	TON (TOF) (h^−1^)	m. p. (°C) (ref.)
1	3-OH	Me	–H	3	89	350.39 (116.79)	180–182^21^
2	3-OH	Me	–Cl	4	88	346.45 (86.61)	240–244^26^
3	3-OH	Ph	–H	2	90	354.33 (177.16)	247–249^22^
4	3-OH	propyl	–H	3.5	87	342.51 (97.86)	129–132^26^
5	3-OH	Cyclopentyl	–H	5	81	318.89 (63.77)	246–248^26^
6	3-OH	Et	–H	4	78	307.08 (76.77)	170–171
7	3-OH	CF_3_	–H	4	80	314.96 (78.74)	177–180
8	3,5-(OH)_2_	Me	–H	4	85	334.64 (83.66)	286–288^21^
9	3,5-(OH)_2_	Me	–Cl	3	90	354.33 (118.11)	320–322^26^
10	3,5-(OH)_2_	Me	–H	4	86	338.58 (84.64)	233–236^26^
11	3,5-(OH)_2_	Cyclopentyl	–H	5	80	314.96 (62.99)	271–272^26^
12	3,5-(OH)_2_	Et	–H	3	88	346.45 (115.48)	261–264
13	2,3-(OH)_2_	Me	–H	4	78	307.08 (76.77)	242–245^26^
14	1-Naphthol	Me	–H	4	75	295.27 (73.81)	154–156^22^

Reaction conditions: phenols (1 mmol), *β*-ketoesters (1 mmol), CNF/T (0.05 g), solvent-free, temperature-110 °C.

^a^Isolated yield.

The sulfur in taurine is the active site of the catalyst. According to EDX data, the amount of S (Sulfur) in the catalyst is 16.28%. Here, we have used 0.05 g of catalyst for 1 mmol of substrate for the synthesis of coumarin. Therefore, 0.05 g of catalyst contains 8.1 × 10^–3^ g of S and is equal to 0.254 mmol of S. Therefore, the TON and TOF of the catalyst are equal to 350.39 and 116.79 h^−1^, respectively.

In the next study, the catalytic activity of CNF/T nano-catalyst for the synthesis of tetra-substituted imidazoles was investigated. Thus, the model reaction of benzil, 4-chlorobenzaldehyde, ammonium acetate, and benzylamine was selected in the presence of CNF/T under various conditions (Table 5).

**Table 5:** Optimization of the reaction conditions for the synthesis of 1,2,4,5- tetra-substituted imidazole.


Entry	Conditions	Time (min)	Yield (%)^a^
	Solvent/temp. (°C)/catalyst (g)		
1	–/80/CNF/T (0.005)	300	Trace
2	–/80/CNF/T (0.01)	150	54
3	–/80/CNF/T (0.015)	125	62
4	–/80/CNF/T (0.02)	90	75
5	–/80/CNF/T (0.025)	60	87
6	–/80/CNF/T (0.03)	50	89
7	–/80/CNF/T (0.035)	45	84
8	–/80/CNF/T/(0.04)	45	86
9	–/50/CNF/T (0.03)	240	30
11	–/90/CNF/T (0.03)	90	84
12	–/100/CNF/T (0.03)	60	74
**14**	**EtOH/80/CNF/T (0.03)**	**25**	**93**
15	EtOH: H_2_O (1:1)/80/CNF/T (0.03)	45	90
16	H_2_O/80/CNF/T (0.03)	90	84
18	–/80/–	300	Trace

Reaction conditions: benzil (1 mmol), 4-chlorobenzaldehyde (1 mmol), ammonium acetate (1 mmol) benzylamine (1 mmol), catalyst (0.03 g), solvent (10 ml).

Significant values are given in bold.

^a^Isolated yield.

To further investigate the application range of CNF/T nano-catalyst, we have employed aromatic aldehyde derivatives in the reaction. The results are shown in Table 6.

**Table 6:** CNF/T-catalyzed the synthesis of 1,2,4,5- tetra-substituted imidazole.


Entry	Ar	R	Time (min)	Yield (%)^a^	TON (TOF) (min^−1^)	m. p. (ref.)
1	4-Cl-C_6_H_4_-	C_6_H_5_CH_2_-	25	93	611.84 (24.47)	161–163^30^
2	2-Cl-C_6_H_4_-	C_6_H_5_CH_2_-	50	87	572.36 (11.44)	140–142^43^
3	4-Me-C_6_H_4_-	C_6_H_5_CH_2_-	60	92	605.26 (10.08)	165–167^30^
4	4-Me-C_6_H_4_-	C_6_H_11_-	90	78	513.15 (5.70)	162–163^43^
5	C_6_H_5_-	C_6_H_11_-	45	89	585.52 (13.01)	170–172^43^
6	3-NO_2_-C_6_H_4_	C_6_H_5_-	25	91	598.68 (23.94)	251–253^43^
7	4-Cl-C_6_H_4_-	C_6_H_5_-	20	90	592.10 (29.60)	150–152^44^
8	4-Me-C_6_H_4_-	C_6_H_5_-	30	88	578.94 (19.28)	186–188^44^
9	C_6_H_5_-	C_6_H_5_-	25	92	605.26 (24.21)	215–218^43^
10	2,6-(Cl)_2_-C_6_H_3_-	C_6_H_11_-	60	88	578.94 (9.64)	139–141^43^

Reaction conditions: benzil (1 mmol), aromatic aldehyde (1 mmol), ammonium acetate (1 mmol) primary amines (1 mmol), CNF/T (0.03 g), EtOH solvent (10 mL), Temperature-80 °C.

^a^Isolated yield.

Here, 0.03 g of catalyst has been used for the synthesis of 1,2,4,5-tetra-substituted imidazoles per 1 mmol of substrate, in this case, 0.03 g of catalyst contains 4.8 × 10^–3^ g of Sulfur and This is equal to 0.152 mmol of S. The TON and TOF for the model reaction are 611.84 and 24.47 min^−1^, respectively.

### A proposed mechanism for the synthesis of coumarin via Pechmann condensation

**Figure 11:**
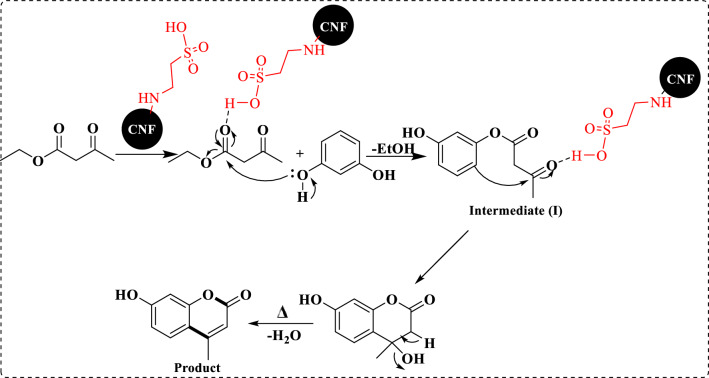
A plausible mechanism for coumarin synthesis by Pechmann condensation.

According to the literature^45^, the synthetic pathway of coumarin through Pechmann condensation is shown in Fig. 11. As can be seen, initially, the reaction is activated by the nucleophilic attack of the hydroxyl group of phenol on ethyl acetoacetate, which leads to the formation of an intermediate (I). The intermediate (I) rapidly gives the product via Brønsted acid-catalyzed intramolecular cleavage followed by dehydration.

### A plausible mechanism for the synthesis of tetra-substituted imidazoles

**Figure 12:**
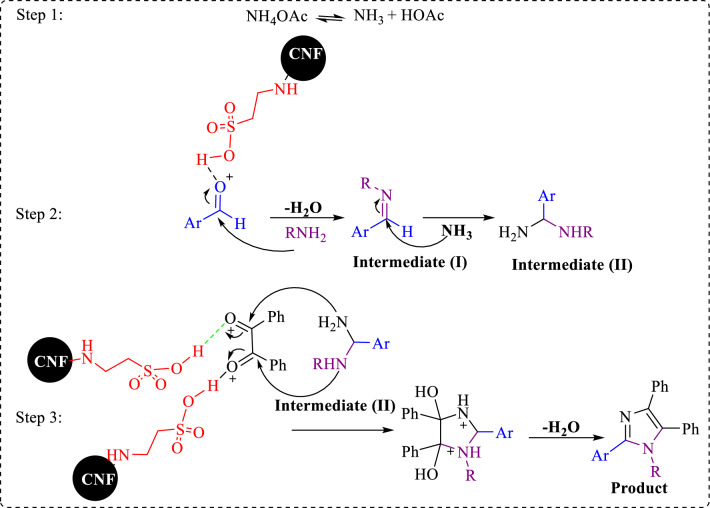
The proposed reaction mechanism for the synthesis of 1,2,4,5- tetra-substituted imidazoles.

The proposed mechanism for the synthesis of 1,2,4,5-tetra-substituted imidazoles is given in Fig. 12^28^. In the first step, CNF/T activates the carbonyl group of aldehyde which reacts with the amine to form intermediate (I). The next step, the intermediate of I, reacts with NH_3_ to form intermediate (II). By condensation of intermediate (II) with 1,2-diketone, followed by dehydration, 1,2,4,5- tetra-substituted imidazole is formed.

To Show the merit of CNF/T nano-catalyst compared to other catalysts for the synthesis of coumarin and tetra-substituted imidazole derivatives a summary of the results was reported in Tables 7 and 8 respectively. The data show that the CNF/T acts as an effective catalyst. As shown in Tables 7 and 8, the CNF/T nano-catalyst works with a relatively high catalytic activity in a short reaction time. Other advantages of this nano-catalyst include its easy preparation as well as easy separation, and recycling.

**Table 7:** Comparison of CNF/T nano-catalyst with other catalysts for the synthesis of coumarin.


Entry	Conditions	Time (h)	References
	Temp. (°C ), solvent, catalyst		
1	110 (MW), no solvent, FeF_3_ (0.05 g)	0.11	^46^
2	150, no solvent, m-ZrP (10 wt %)	4	^47^
3	150, no solvent, Zr-TMS (0.1 g)	20	^48^
4	90, no solvent, MNESA (0.07 g)	1.5	^49^
5	80, no solvent, SMA NPs (5 mol %)	0.33	^50^
6	110, no solvent, CNF/T (0.05 g)	3	This work

**Table 8:** Comparison activity of CNF/T nano-catalyst with other catalysts for the synthesis of imidazole derivatives.


Entry	Conditions	Time (min)	References
	Temp. (°C), solvent, catalyst		
1	140, no solvent, SBPPSA (0.25 g)	60	^51^
2	r. t., EtOH, Fe_3_O_4_@PVA-SO_3_H (0.05 g)	40	^52^
3	100, no solvent, HNO_3_@nano SiO_2_ (0.012 g)	225	^53^
4	110, no solvent, nano- TiCl_4_.SiO_2_ (0.1 g)	25	^43^
5	80, no solvent, SiO_2_:SnO_2_ (0.5 g)	30	^44^
6	125, no solvent, pyridinium hydrogen sulfate (4 mmol)	25	^34^
7	120, toluene, Cu_0.9_Fe_0.1_@RCAC (0.05g)	120	^35^
8	80, EtOH, CNF/T (0.03 g)	25	This work

### Catalyst reusing

**Figure 13:**
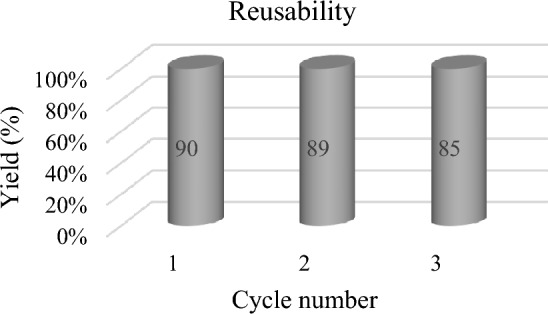
The reusing of CNF/T for coumarin synthesis by Pechmann condensation.

The reusing study of CNF/T nano-catalyst for the synthesis of coumarin is given in Fig. 13. The model reaction was carried out under optimal conditions for Pechmann condensation.

In repeated experiments, the catalyst was washed with ethanol, dried at room temperature, and used without activation. The results of the experiments showed that the catalytic activity of the nano-catalyst was slightly reduced which is probably due to the interaction of the acidic hydrogen of taurine in the nano-catalyst with the hydroxyl group of phenol as a nucleophile (90–85%).

**Figure 14:**
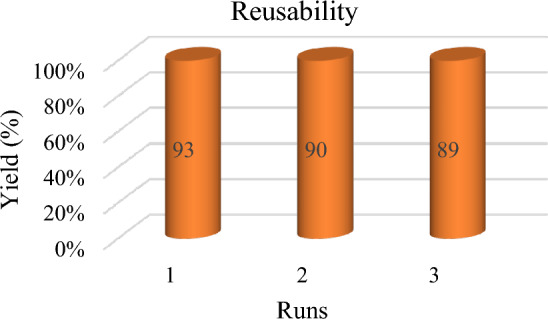
Reusability study of CNF/T for the synthesis of 1,2,4,5- tetra-substituted imidazoles.

To determine the reusing application of CNF/T for the synthesis of 1,2,4,5- tetra-substituted imidazoles, at the end of the reaction, the catalyst was separated from the reaction mixture using filtration. Then it was washed with ethanol to remove the remaining product. It was dried at ambient temperature and reused in the next reaction with excellent yield (Fig. 14). According to Fig. 14, after reusing the catalyst three times, there is no significant decrease in product yield.

### Leaching test of CNF/T

**Figure 15:**
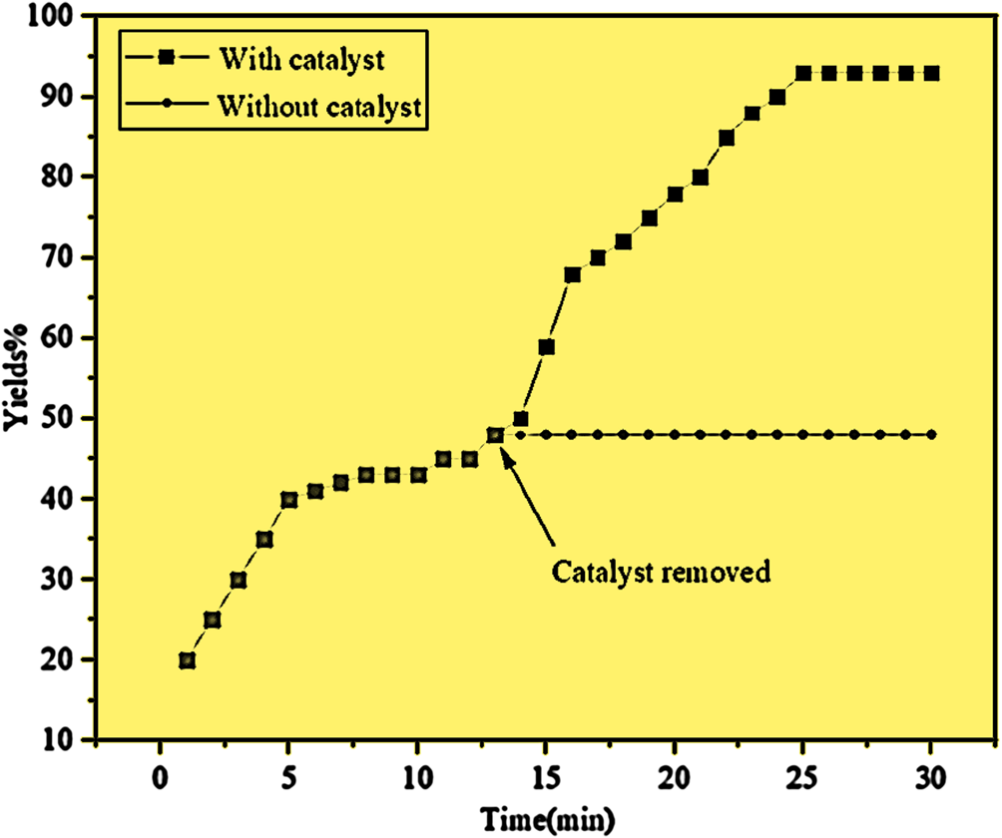
Catalyst leaching test for CNF/T.

To investigate the leakage of nano-catalyst, it was suggested that the model reactions be carried out in the presence of CNF/T nano-catalyst. Then after half of the reaction time, the nano-catalyst was separated from the reaction mixture and the reaction continued without the presence of the nano-catalyst. As evident in Fig. 15, no reaction progress was achieved in the absence of a nano-catalyst, which indicates that the nano-catalyst did not leak into the reaction mixture.

**Figure 16:**
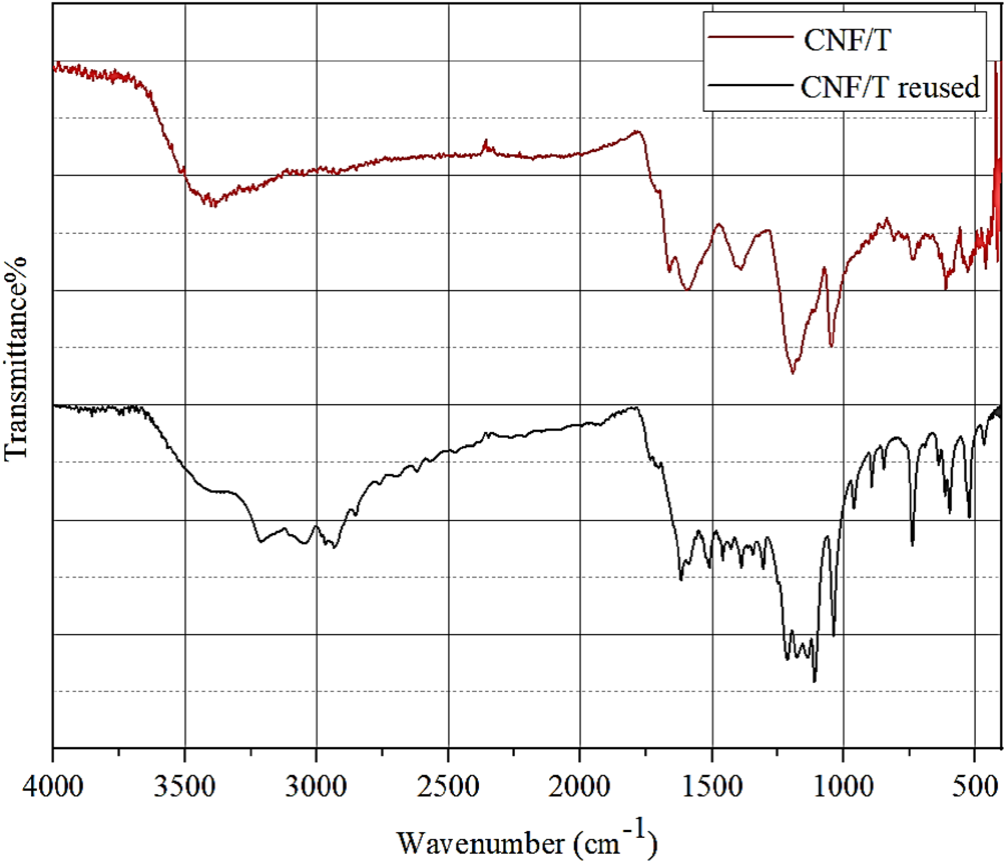
FT-IR of recovered CNF/T.

The FT-IR Spectrum of the recovered CNF/T nano-catalyst was performed after the third run. As can be seen according to the obtained spectrum of FT-IR and comparing it with the primary nano-catalyst, it shows that the nano-catalyst has preserved its structure (Fig. 16).

## Experimental section

### Materials and methods

Chemicals were purchased from Merck, Fluka, and Aldrich Chemical Companies. ^1^H NMR and ^13^C NMR spectra were recorded at 400 and 100 MHz, respectively. Fourier transform infrared (FT-IR) measurements (in KBr pellets or ATR) were recorded on a Bruker spectrometer. Melting points were determined on a Büchi B-540 apparatus. The X-ray diffraction (XRD) pattern was obtained by a Philips Xpert MPD diffractometer equipped with a Cu Kα anode (*k* = 1.54 Å) in the 2*θ* range from 10 to 80°. Field Emission Scanning Electron Microscopy (FESEM) was obtained on a Mira 3-XMU. VSM measurements were performed by using a vibrating sample magnetometer (Meghnatis Daghigh Kavir Co. Kashan Kavir, Iran). Energy-dispersive X-ray spectroscopy (EDS) of nano-catalyst was measured by an EDS instrument and Phenom pro-X. The EDX-MAP micrographs were obtained on the MIRA II detector SAMX (France). Thermal gravimetric analysis (TGA) was conducted using the “STA 504” instrument. Transmission electron microscopy (TEM) was obtained using a Philips CM120 with a LaB6 cathode and an accelerating voltage of 120 kV. BELSORP MINI II nitrogen adsorption apparatus (Japan) for recording Brunauer–Emmett–Teller (BET) of nano-catalyst at 77 K. UV–Vis spectroscopy was measured using the Analytical Jena. X-ray Photoelectron Spectroscopy (XPS) analysis was done with BESTEC (EA 10).

### CNF/T preparation

To prepare the CNF/T, three steps including oxidation, acylation, and then amination were performed. In the first, the carbon nanofiber (CNF) was rinsed with HCl (0.1 M) and NaOH (0.1 M) solutions to remove alkaline and acidic impurities, respectively. The washed CNF was then treated for 50 h in boiling concentrated HNO_3_ (68%) in a reflux condenser to remove amorphous carbon and form oxidized carbon nanofiber (OX-CNF). The OX-CNF was sequentially washed with deionized water, then with ammonia (NH_4_OH), water, and HCl, and again with deionized water until the pH was stabilized. In the next step, in a round bottom flask, the mixture of 0.11 g of OX-CNF with 15 mL of thionyl chloride and 1 mL of DMF refluxed at 80 °C for 2 h to form carbon nanofiber containing acid chloride functional groups (CNF-Cl). Excess thionyl chloride was evaporated using a vacuum and the remaining mixture was washed with dichloromethane (CH_2_Cl_2_). In the last step, first, in a round bottom flask, 1 mmol of taurine and 1 mmol of sodium acetate were mixed in 20 mL of DMF solvent for 20 min at a temperature of 120 °C in a reflux condenser. Then 1 g of CNF-Cl was added to the mixture and refluxed for 24 h. After that, it was washed with CH_2_Cl_2_. As a result, a black solid of CNF/T was prepared.

### Synthesis of coumarin derivatives via Pechmann condensation

In a round bottom flask, the mixture of resorcinol (1 mmol, 0.11 g), ethyl acetoacetate (1 mmol, 0.13 g), and CNF/T (0.05 g) was heated at 110 °C for the appropriate time. The progress of the reaction was monitored by thin-layer chromatography (n-hexane: ethyl acetate 4:1). After the completion of the reaction, the reaction mixture was dissolved in hot ethanol and the catalyst was separated by filtration. The solvent was then removed under reduced pressure and the resulting crude product was purified by recrystallization using ethanol.

### Synthesis of tetra-substituted imidazole derivatives

For the synthesis of 1,2,4,5-tetra-substituted imidazoles, the reaction between benzil, aldehyde, ammonium acetate, and benzylamine was carried out in the presence of the CNF/T catalyst under ethanol reflux conditions. For this purpose, in a round bottom flask, the mixture of benzil (1 mmol, 0.21 g), aldehyde (1 mmol), ammonium acetate (1 mmol, 0.077 g), benzylamine (1 mmol, 0.107 g), and CNF/T (0.03 g) refluxed in 10 ml of ethanol. After the completion of the reaction (TLC n-hexane: ethyl acetate 7:3) the CNF/T catalyst was separated from the reaction mixture using filtration and then cold water was added to the reaction mixture and the product was separated by filtration.

## Conclusion

In summary, we have successfully prepared a CNF/T nano-catalyst via three steps: acid treatment, acylation, and amination. CNF/T is stable, biocompatible, and cost-effective with good acidic properties. The structure of the nano-catalyst was successfully confirmed using FT-IR, TGA, FESEM, XRD, TEM, EDX, EDS-MAP, BET, and XPS techniques. The Hammett acidity function of the nano-catalyst was measured as 1.47 for 5 × 10^–2^ mg/mL of CNF/T in 0.3 mM of 4-nitroaniline using UV–Vis spectroscopy. CNF/T nano-catalyst shows high catalytic activity during the reaction of coumarin and 1,2,4,5-tetra substituted imidazoles. Also, according to the leaching test and the FT-IR spectrum of the recovered catalyst show that the CNF/T nano-catalyst has considerable stability. We believe that the modification of the surface of CNF by taurine and then using them for the synthesis of heterocyclic compounds such as coumarin and tetra-substituted imidazoles is an effective and practical tool to prepare a suitable catalytic system (Supplementary Information S1).

### Supplementary Information

Supplementary Information.

## Data Availability

All data generated or analyzed during this study are included in this published article.
