# Supplementary material for: Carbon nanofiber/taurine-catalyzed synthesis of coumarin and 1,2,4,5-tetra-substituted imidazole derivatives under metal-free conditions
Source: Sci Rep. 2024 May 9;14:10677. doi: 10.1038/s41598-024-61249-2 (PMC11082250; doi:10.1038/s41598-024-61249-2)
Supplement: Supplementary file 1 — Supplementary Information. [file 41598_2024_61249_MOESM1_ESM.docx]

**Carbon nanofiber/taurine -Catalyzed synthesis of coumarin and 1,2,4,5-tetra-substituted imidazole derivatives under metal-free conditions**

Dina Mallah^1^, Bi Bi Fatemeh Mirjalili*^1^, Abdolhamid Bamoniri^2^

^1^Department of Chemistry, College of Science, Yazd University, P.O. Box 89195-741, Yazd, I.R.IRAN. Fax: +983538210644; Tel: +983531232672. Email: [fmirjalili@yazd.ac.ir](mailto:fmirjalili@yazd.ac.ir)

^2^Department of Organic Chemistry, Faculty of Chemistry, University of Kashan, Kashan, I.R.IRAN.

**7-Hydroxy-4-methyl-2*H*-chromene-2-one (*Table 3, entry 1*)**

White solid, m.p.180-182 °C. FT-IR (ATR) ῡ (cm^-1^): 3225, 1704, 1670, 1606, 1565, 1360, 1323, 1140, 1070, 982, 851, 807. ^1^HNMR (Acetone-*d_6_*, 400 MHz)/ δ (ppm): OH is unobserved, 7.61 (d, *J* = 8.8 Hz, 1H), 6.85 (dd, *J* = 8.0.Hz, *J* = 2.0 Hz, 1H), 6.74 (s, 1H), 6.08 (s, 1H), 2.41 (s, 3H).

**The FT-IR of 7-Hydroxy-4-methyl-2*H*-chromene-2-one**

**The ^1^H NMR spectrum of 7-Hydroxy-4-methyl-2*H*-chromene-2-one**

**3-Chloro-7-hydroxy-4-methyl-2*H*-chromene-2-one (*Table 3, entry 2*)**

Pale orange solid, m.p. 240-244 °C. FT-IR (ATR) ῡ (cm^-1^): 3297, 1682, 1602, 1559, 1379, 1261, 1209, 1144, 1076, 849, 784, 754. ^1^HNMR (Acetone-*d_6_*, 400 MHz)/ δ (ppm): OH is unobserved, 7.67 (d, *J* = 8.8 Hz, 1H), 6.90 (dd, *J* = 8.8 Hz, *J* = 2.4 Hz, 1H), 6.77 (d, *J* = 2.4 Hz, 1H), 2.55 (s, 3H).


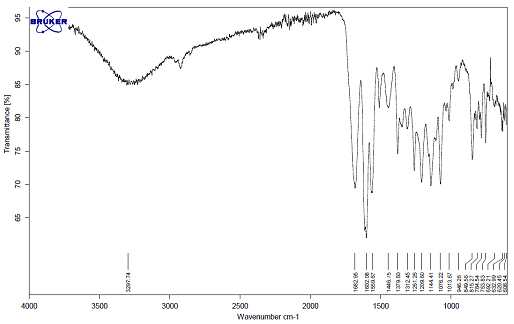


**The FT-IR of 3-Chloro-7-hydroxy-4-methyl-2*H*-chromene-2-one**

**The ^1^H NMR spectrum of 3-Chloro-7-hydroxy-4-methyl-2*H*-chromene-2-one**

**7-Hydroxy-4-phenyl-2*H*-chromene-2-one (*Table 3, entry 3*)**

Pale pink solid, m.p. 247-249 °C. FT-IR (ATR) ῡ (cm^-1^): 3076, 2927, 1681, 1603, 1450, 1371, 12306, 1212, 1147, 1001, 846, 758, 687, 596.

**The FT-IR of 7-Hydroxy-4-phenyl-2*H*-chromene-2-one**

**7-Hydroxy-4-propyl-2*H*-chromene-2-one (*Table 3, entry 4*)**

Light brown solid, m.p. 129-132 °C. FT-IR (ATR) ῡ (cm^-1^): 3398, 2965, 2875, 1675, 1599, 1454, 1396, 1271, 1161, 1135, 1002, 895, 837, 809, 746. ^1^HNMR (Acetone-*d_6_*, 400 MHz)/ δ (ppm): 9.66 (s, 1H), 7.65 (d, *J* = 8.8 Hz, 1H), 6.84 (dd, *J* = 8.8 Hz, *J* = 2.4 Hz, 1H), 6.74 (d, *J* = 2.4 Hz, 1H), 6.05 (s,1H), 2.75 (t, *J* = 7.6 Hz, 2H), 1.70 (m, 2H), 1.00 (t, *J* = 7.6 Hz, 3H).

**The FT-IR of 7-Hydroxy-4-propyl-2*H*-chromene-2-one**

**7-Hydroxy-2,3-dihydrocyclopenta[*c*]-chromene-4(1*H*) -one (*Table 3, entry 5*)**

Cream solid, m.p. 246-248 °C. FT-IR (ATR) ῡ (cm^-1^): 3204, 2952, 1670, 1619, 1560, 1518, 1392, 1307, 1265, 1217, 1148, 1132, 1074, 968, 851, 827, 754, 709. ^1^HNMR (Acetone-*d_6_*, 400 MHz)/ δ (ppm): 9.32 (s, 1H), 7.41 (d, *J* = 8.4 Hz, 1H), 6.83 (dd, *J* = 8.8 Hz, *J* = 2.4 Hz, 1H), 6.77 (d, *J* = 2.4 Hz, 1H), 3.06 (t, *J* = 7.6 Hz, 2H), 2.75 (t, *J* = 6 Hz, 2H), 2.15 (quintet, *J* = 7.6 Hz, 2H).

**The FT-IR of 7-Hydroxy-2,3-dihydrocyclopenta[*c*]-chromene-4(1*H*) -one**

**The ^1^H NMR spectrum of 7-Hydroxy-2,3-dihydrocyclopenta[*c*]-chromene-4(1*H*) -one**

**5,7-Dihydroxy-4-methyl-2*H*-chromene-2-one (*Table 3, entry 6*)**

Cream solid, m.p. 286-288 °C. FT-IR (ATR) ῡ (cm^-1^): 3434, 3197, 2921, 1670, 1622, 1557, 1384, 1365, 1302, 1162, 1079, 828. ^1^HNMR (Acetone-*d_6_*, 400 MHz)/ δ (ppm): 9.4 (sbr, 1H), 9.2 (sbr, 1H), 6.2–6.4 (d, 2H), 5.83 (s, 1H), 2.55 (s, 3H).

**The FT-IR of 5,7-Dihydroxy-4-methyl-2*H*-chromene-2-one**

**The ^1^H NMR spectrum of 5,7-Dihydroxy-4-methyl-2*H*-chromene-2-one**

**3-Chloro-5,7-dihydroxy-4-methyl-2*H*-chromene-2-one (*Table 3, entry 7*)**

Light brown solid, m.p. 320-322 °C. FT-IR (ATR) ῡ (cm^-1^): 3156, 1681, 1627, 1541, 1462, 1359, 1280, 1173, 1091, 1023, 756, 563**.** ^1^HNMR (Acetone-*d_6_*, 400 MHz)/ δ (ppm): 10.78 (s, 1H), 10.45 (sbr, 1H), 6.32 (s, 1H), 6.20 (s, 1H), 2.68 (s, 3H).

**The FT-IR of 3-Chloro-5,7-dihydroxy-4-methyl-2*H*-chromene-2-one**

**The ^1^H NMR spectrum of 3-Chloro-5,7-dihydroxy-4-methyl-2*H*-chromene-2-one**

**5,7-Dihydroxy4-propyl-2*H*-chromene-2-one (*Table 3, entry 8*)**

Cream solid, m.p. 233-236 °C. FT-IR (ATR) ῡ (cm^-1^): 3203, 2965, 1623, 1594, 1394, 1288, 1224, 1158, 1099, 828. ^1^HNMR (Acetone-*d_6_*, 400 MHz)/ δ (ppm): 10.67 (s, 1H), 10.38 (s, 1H), 6.35 (d, *J =* 2.4 Hz, 1H), 6.26 (d, *J =* 2.0 Hz, 1H), 5.91 (s, 1H), 2.93 (t, *J =* 7.2 Hz, 2H), 1.67 (sextet, *J =* 7.6 Hz, 2H), 1.03 (t, *J =* 7.2 Hz, 3H).

**The FT-IR of 5,7-Dihydroxy4-propyl-2*H*-chromene-2-one**

**The ^1^H NMR spectrum of 5,7-Dihydroxy4-propyl-2*H*-chromene-2-one**

**The ^1^H NMR spectrum of 5,7-Dihydroxy4-propyl-2*H*-chromene-2-one**

**7,9-Dihydroxy-2,3-dihydrocyclopenta[*c*]-chromene-4(1*H*)-one (*Table 3, entry 9*)**

Pale yellow solid, m.p. 271-272 °C. FT-IR (ATR) ῡ (cm^-1^): 3227, 2967, 1680, 1655, 1625, 1559, 1466, 1397, 1286, 1154, 1113, 1082, 840, 740. ^1^HNMR (Acetone-*d_6_*, 400 MHz)/ δ (ppm): 10.55 (sbr, 1H), 10.35 (sbr, 1H), 6.39 (d, *J =* 2.0 Hz, 1H), 6.35 (d, *J =* 2.0 Hz, 1H), 3.37 (t, *J =* 7.6 Hz, 2H), 2.75 (t, *J =* 7.6 Hz, 2H), 2.14 (quintet, *J =* 7.6 Hz, 2H). ^13^C NMR (DMSO-*d_6_*, 100 MHz)/ δ (ppm): 160.6, 159.4, 156.5, 156.4, 156.3, 125.0, 120.1, 98.5, 94.1, 35.6, 28.9, 22.3.

**The FT-IR of 7,9-Dihydroxy-2,3-dihydrocyclopenta[*c*]-chromene-4(1*H*)-one**

**The ^13^C NMR spectrum of 7,9-Dihydroxy-2,3-dihydrocyclopenta[*c*]-chromene-4(1*H*)-one**

**The ^1^H NMR spectrum of 7,9-Dihydroxy-2,3-dihydrocyclopenta[*c*]-chromene-4(1*H*)-one**

**The ^1^H NMR spectrum of 7,9-Dihydroxy-2,3-dihydrocyclopenta[*c*]-chromene-4(1*H*)-one**

**7,8-Dihydroxy4-methyl-2*H*-chromene-2-one (*Table 3, entry 10*)**

White solid, m.p. 242-245 °C. FT-IR (ATR) ῡ (cm^-1^): 3413, 3230, 1648, 1620, 1600, 1583, 1385, 1308, 1188, 1062, 1007, 863, 804, 766, 723.

**The FT-IR of 7,8-Dihydroxy4-methyl-2*H*-chromene-2-one**

**4-Methyl-2*H*-benzo[*h*] chromene-2-one (*Table 3, entry 11*)**

Cream solid, m.p. 154-156 °C. FT-IR (ATR) ῡ (cm^-1^): 2960, 1710, 1639, 1611, 1561, 1473, 1375, 1339, 1270, 1237, 1172, 1082, 1028, 944, 867, 842, 809, 748. ^1^HNMR (Acetone-*d_6_*, 400 MHz)/ δ (ppm): OH is unobserved. 7.83 (d, *J* = 7.6.Hz, 1H), 7.70–7.73 (m, 5H), 6.42 (s, 1H), 2.59 (s, 3H).

**The FT-IR of 4-Methyl-2*H*-benzo[*h*] chromene-2-one**

**The ^1^H NMR spectrum of 4-Methyl-2*H*-benzo[*h*] chromene-2-one**

1-Benzyl-2-(4-chloro phenyl)-4,5-diphenyl-imidazole (*Table 5, entry 1*)

white solid, m.p. 161-163 ^°^C. FT IR (ATR) ῡ = 3062, 1601 (C=C stretch), 1479 (C=N stretch), 1089 (C-Cl stretch), 835 ( C-H bend), 691 ( C-H bend) cm^-1^. ^1^H NMR (500 MHz, CDCl_3_): 5.13 (s, 2H), 6.85 (m, 2H), 7.2 (t,  *J*=7.2Hz, 1H), 7.27-7.3 (m, 7H), 7.3-7.4 (m, 3H), 7.4 (d*, J*=6.7Hz, 2H), 7.62 (d, *J*=7.3Hz, 2H), 7.64 (d, *J*=6.7, 2H) ppm.

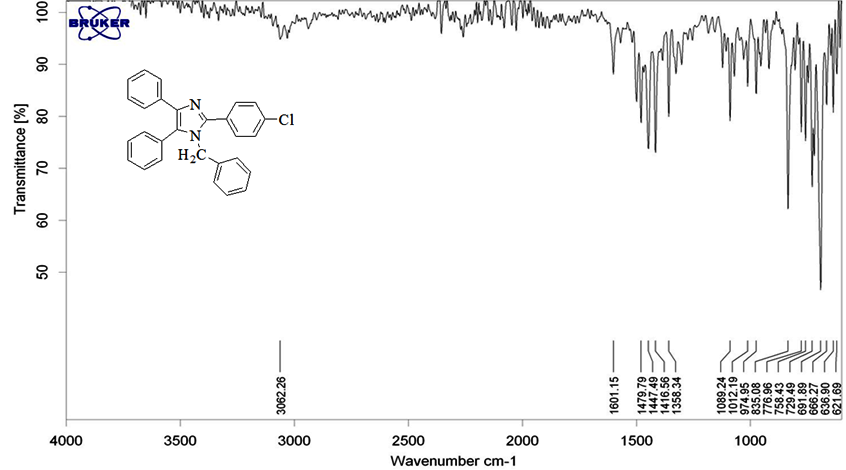


**The FT-IR of 1-Benzyl-2-(4-chloro phenyl)-4,5-diphenyl-imidazole**


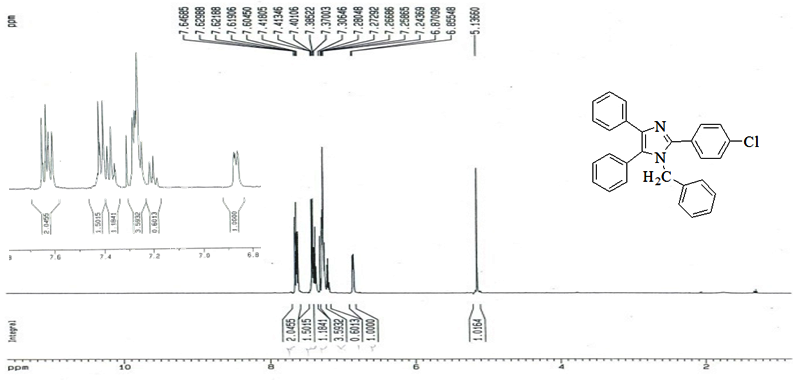


**The ^1^H NMR spectrum of 1-Benzyl-2-(4-chloro phenyl)-4,5-diphenyl-imidazole**

**1-Benzyl-2-(2-chlorophenyl)-4,5-diphenyl-imidazole (*Table 5, entry 2*)**

white solid, m.p. 140-142 °C. FT-IR (KBr) ῡ = 3027 (C-H, stretch), 1601 (C=N, stretch), 1477 (C=C stretch),1072 (C-Cl stretch), 799 (C-H bend), 696 (C-H bend) cm^-1^. ^1^H NMR (300 MH,z CDCl_3_/DMSO-d_6_): 4.95 (s, 2H), 6.62-6.65 (m, 2H), 7.07-7.48 (m, 15H), 7.58-7.60 (m, 2H) ppm.

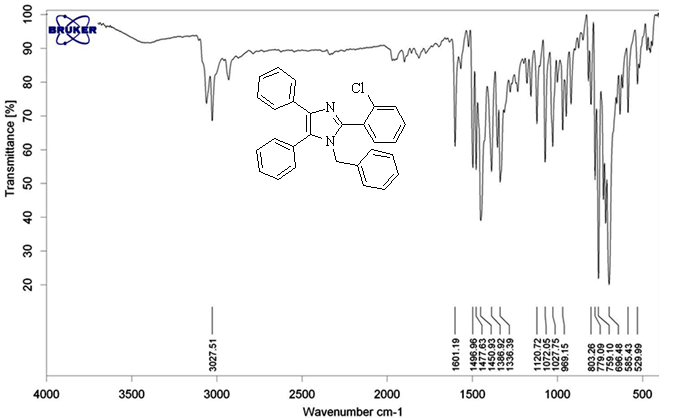


**The FT-IR of 1-Benzyl-2-(2-chlorophenyl)-4,5-diphenyl-imidazole**


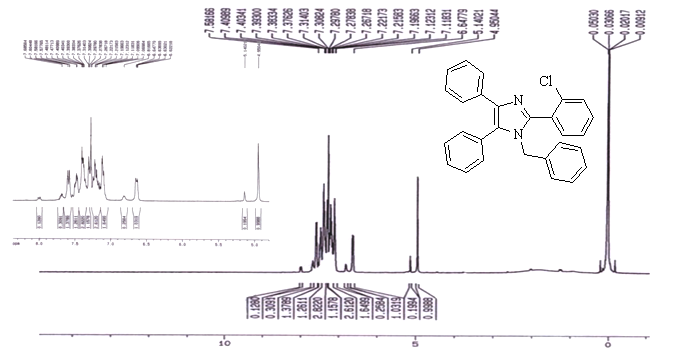


**The ^1^H NMR spectrum of 1-Benzyl-2-(2-chlorophenyl)-4,5-diphenyl-imidazole**

**1-Benzyl-2-(4-methyl phenyl)-4,5-diphenyl-imidazole (*Table 5, entry 3*)**

White solid, m.p. 165-167 ^°^C FT-IR (KBr) ῡ = 3026 (C-H stretch), 1601 (C=N stretch), 1484 (C=C stretch), 827 (C-H bend), 702 ( C-H bend) cm^-1^. ^1^H NMR (500 MHz , CDCl_3_): 2.4 (s, 3H), 5.12 (d, *J*=5.7Hz, 2H), 6.8 (m, 2H), 7.15 (t, *J*= 6.8Hz, 1H), 7.13-7.23 (m, 8H), 7.3-7.36 (m, 4H), 7.56 (d, *J*=7.9Hz, 2H), 7.58 (d, *J*=8.2Hz, 2H) ppm.

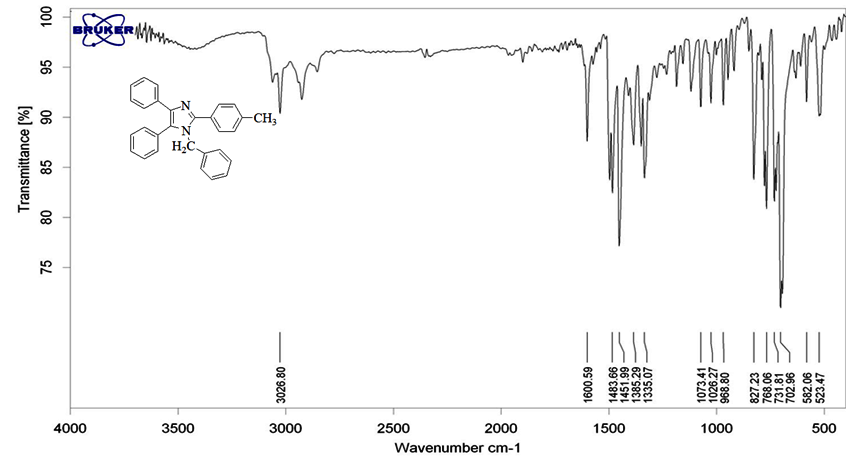


**The FT-IR of 1-Benzyl-2-(4-methyl phenyl)-4,5-diphenyl-imidazole**


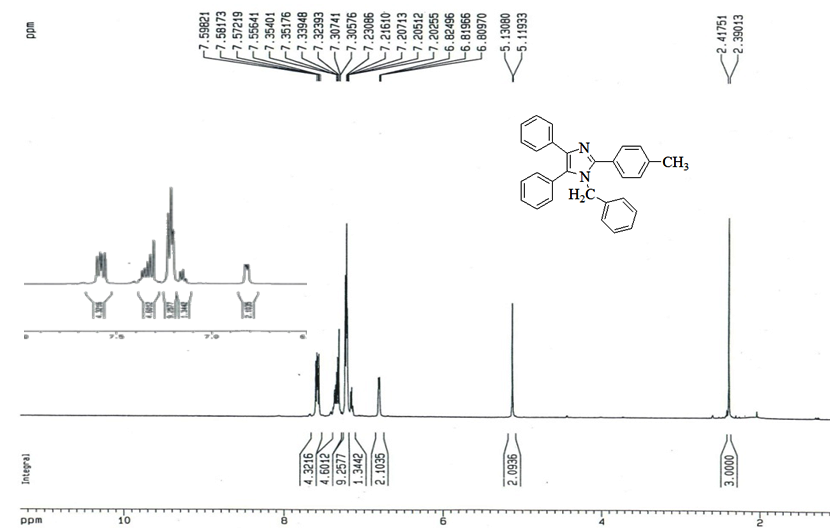


**The ^1^H NMR spectrum of 1-Benzyl-2-(4-methyl phenyl)-4,5-diphenyl-imidazole**

**1-****Cyclohexyl-2-(4-methylphenyl)-4,5-diphenyl-imidazole (*Table 5, entry 4*)**

white solid, m.p. 162-163 ^o^C. FT-IR (KBr) ῡ = 2928 (C-H stretch), 1598 (C=N stretch), 1499 (C=C stretch) _,_ 829 (C-H bend), 693 (C-H bend) cm^-1^. ^1^HNMR (500 MHz ,CDCl_3_): 0.77-1.87 (m, 10H), 2.44 (s, 3H), 3.98 (m, 1H), 7.06-7.53 (m, 14H) ppm.

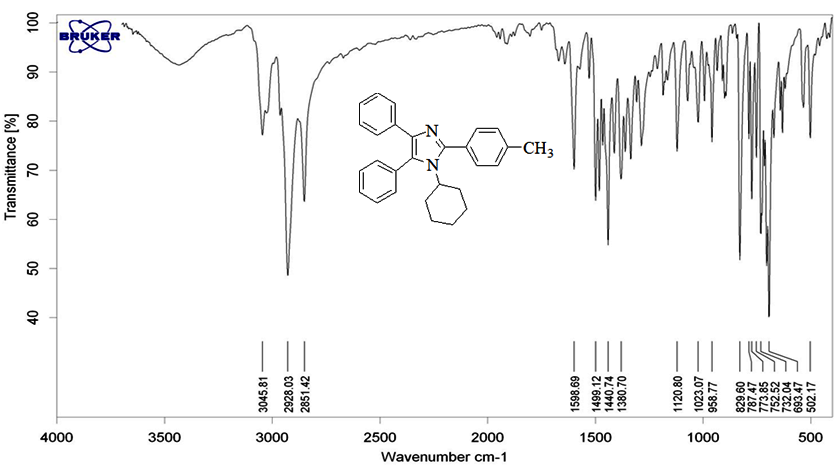


**The FT-IR of 1-Cyclohexyl-2-(4-methylphenyl)-4,5-diphenyl-imidazole**


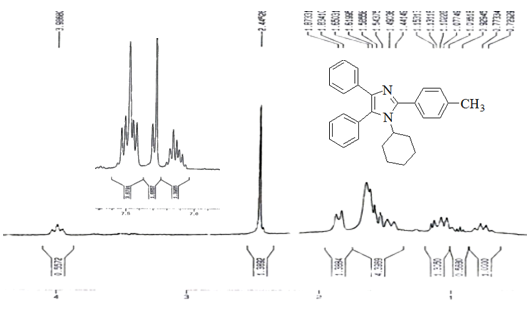


**The ^1^H NMR spectrum of 1-Cyclohexyl-2-(4-methylphenyl)-4,5-diphenyl-imidazole**

**1-Cyclohexyl-2, 4,5-triphenyl-imidazole (*Table5, entry 5*)**

white solid, m.p. 170-172 °C. FT-IR (KBr) ῡ = 3055 (C-H strech), 2931 (C-H stretch), 1601 (C=N stretch), 1442 (C=C stretch), 773 (C-H bend), 700 (C-H bend) cm^-1^. ^1^H NMR (300 MHz ,CDCl_3_): 1.03-1.85 (m, 9H), 3.90 (m, 1H), 7.43 (m, 13H) 7.62-7.64 (m, 2H) ppm.

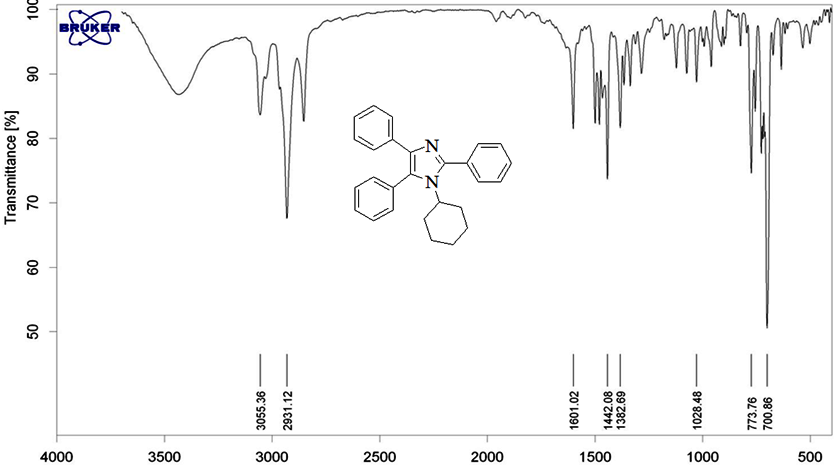


**The FT-IR of 1-Cyclohexyl-2, 4,5-triphenyl-imidazole**


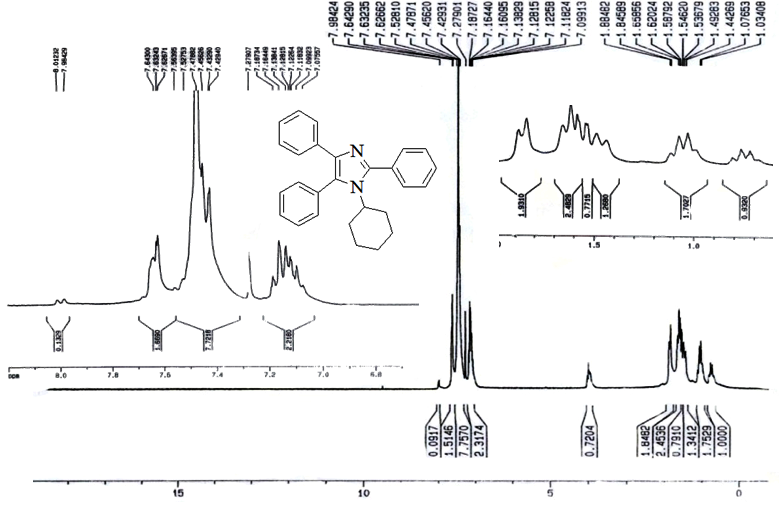


**The ^1^H NMR spectrum of 1-Cyclohexyl-2, 4,5-triphenyl-imidazole**

**2-(3-Nitroro phenyl)-1,4,5-diphenyl-imidazole (*Table 5, entry 6*)**

Yellow solid, m.p. 251-253°C. FT-IR (KBr) ῡ = 1608 (C=N stretch), 1525 (N=O stretch), 1492 (C=C stretch), 1342 (N=O stretch), 695 (C-H bend) cm^-1^. ^1^H NMR (500 MHz ,CDCl_3_/DMSO-d_6_): 7.09 (brd, *J*=7.8Hz, 2H), 7.14 (brd, *J*=7.8Hz, 2H), 7.21-7.27 (m, 6H), 7.3-7.34 (m, 3H), 7.41 (t, *J*=8.2Hz, 1H),7.59 (d, *J*=8.2Hz, 2H), 7.77 (brd, *J*=8.9Hz, 1H), 8.09 (brd, *J*=8.2Hz, 1H),8.26 (brs, 1H) ppm.

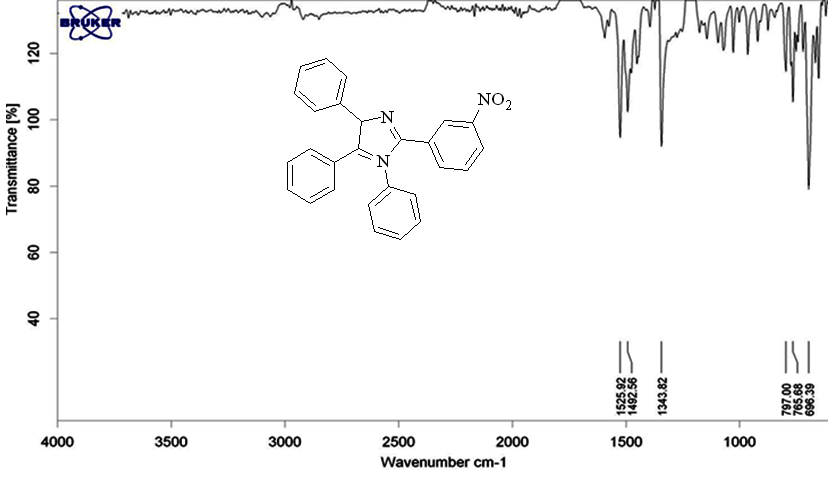


**The FT-IR of 2-(3-Nitroro phenyl)-1,4,5-diphenyl-imidazole**


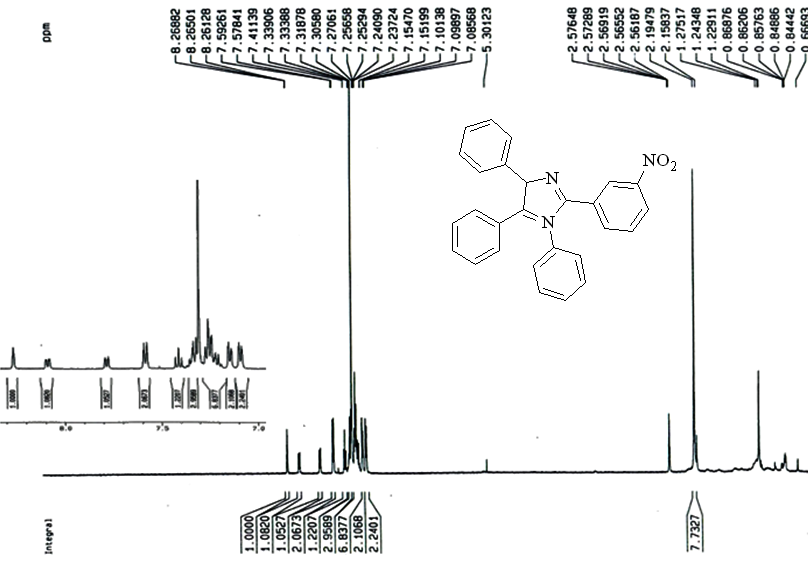


**The ^1^H NMR spectrum of 2-(3-Nitroro phenyl)-1,4,5-diphenyl-imidazole**

2-(4-Chlorolphenyl)-1,4,5-triphenyl-imidazole (*Table 5, entry 7*)

White solid, m.p. 150-152 ^°^C. FT-IR (ATR) ῡ = 1597 (C=N, stretch), 1494 (C=C, stretch), 1084 (C-Cl stretch), 835 ( C-H bend), 718 ( C-H bend) cm^-1^. ^1^H NMR (500 MHz , CDCl_3_): 7.09 (brd, *J*= 8.2Hz, 2H), 7.17 (brd, *J*= 8.15, 2H), 7.24-7.34 (m, 11H), 7.4 (brd, *J*= 8.62, 2H), 7.63 (brd, *J*= 8.6, 2H) ppm.

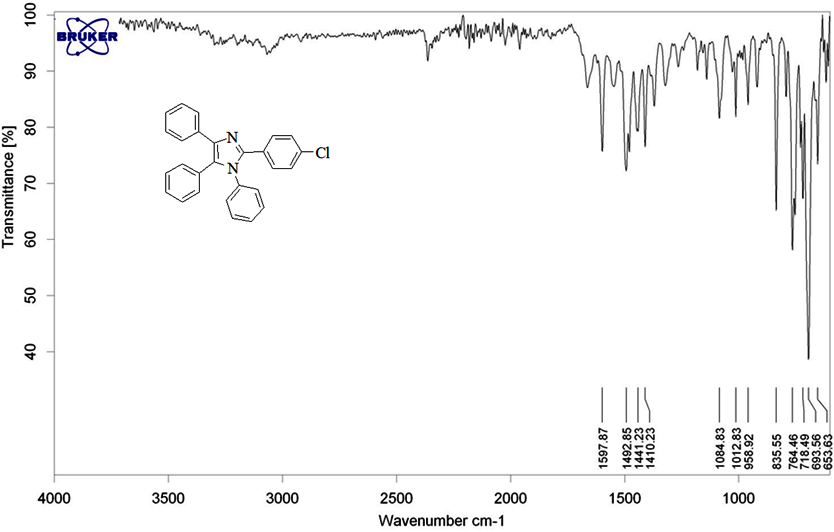


**The FT-IR of 2-(4-Chlorolphenyl)-1,4,5-triphenyl-imidazole**


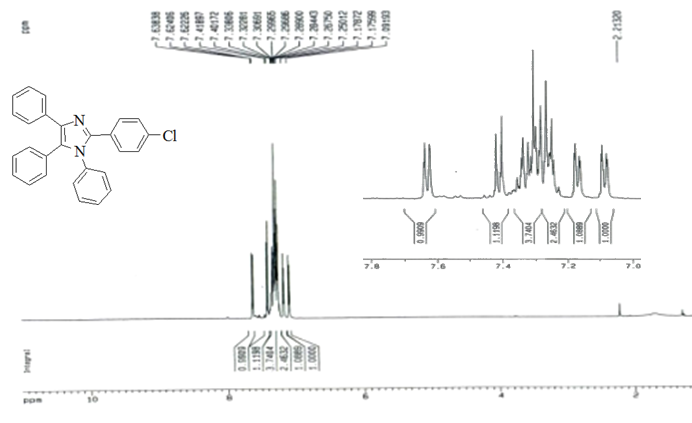


**The ^1^H NMR spectrum of 2-(4-Chlorolphenyl)-1,4,5-triphenyl-imidazole**

2-(4-Methylphenyl)-1,4,5-diphenyl-imidazole (*Table 5, entry 8*)

white solid, m.p. 186-188°C. FT-IR (ATR) ῡ = 1596 (C=N, stretch), 1492 (C=C, stretch), 822 (C-H bend), 693 (C-H bend) cm^-1^. ^1^H NMR (500 MHz, CDCl_3_): 2.35 (s, 3H), 7.08 (brd, 4H) 7.17 (m, 2H), 7.2-7.3 (m, 9H), 7.36 (d, *J*=8.14 Hz, 2H), 7.65 (d, *J*=7.2Hz, 2H) ppm.

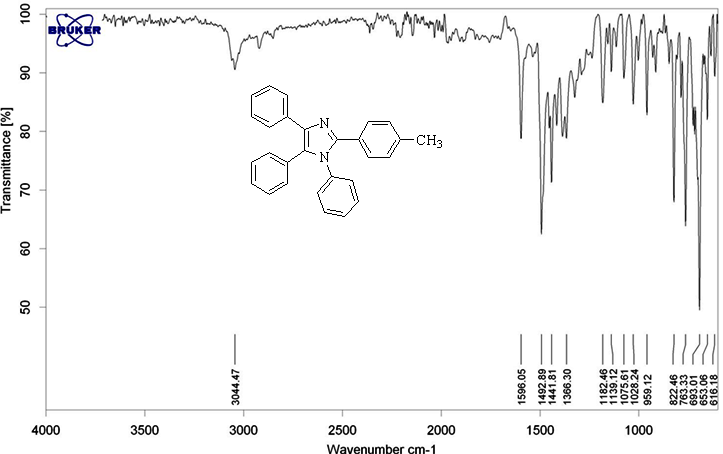


**The FT-IR of 2-(4-Methylphenyl)-1,4,5-diphenyl-imidazole**


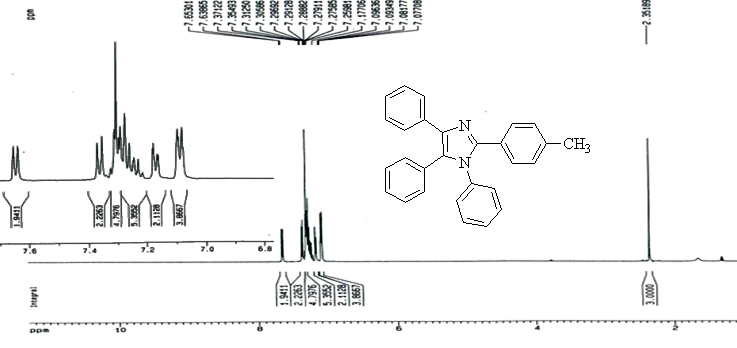


**The ^1^H NMR spectrum of 2-(4-Methylphenyl)-1,4,5-diphenyl-imidazole**

1,4,5-Triphenyl-imidazole (*Table 5, entry 9*)

white solid, m.p. 215-217 ^°^C. FT- IR (KBr) ῡ = 1600 (C=N stretch), 1496 (C=C stretch), 796 (C-H bend), 695 (C-H bend) cm^-1^. ^1^H MNR (400 MHz CDCl_3_): 6.83 (d, *J*= 6.0Hz, 2H), 6.93 (d, *J*= 6.4Hz, 2H), 6.95-7.11 (m, 12H), 7.23 (d, *J*= 5.6Hz, 2H), 7.40 (d, *J*= 7.6Hz, 2H) ppm.

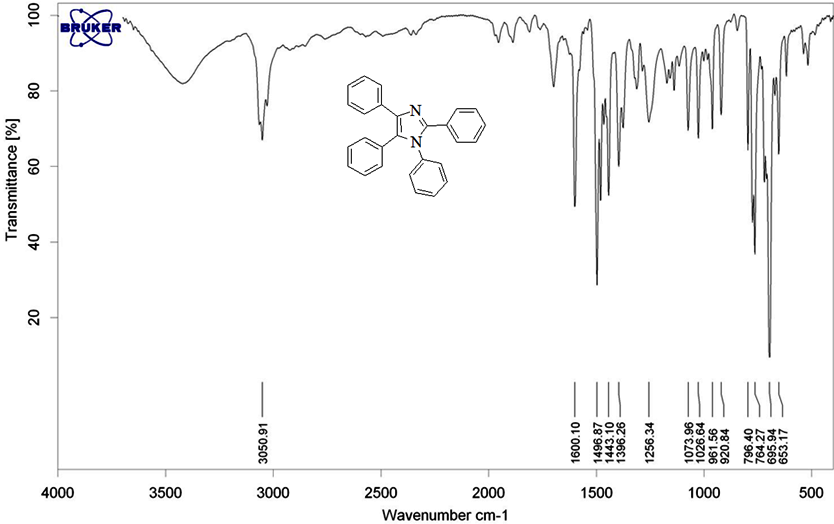


**The FT-IR of 1,4,5-Triphenyl-imidazole**


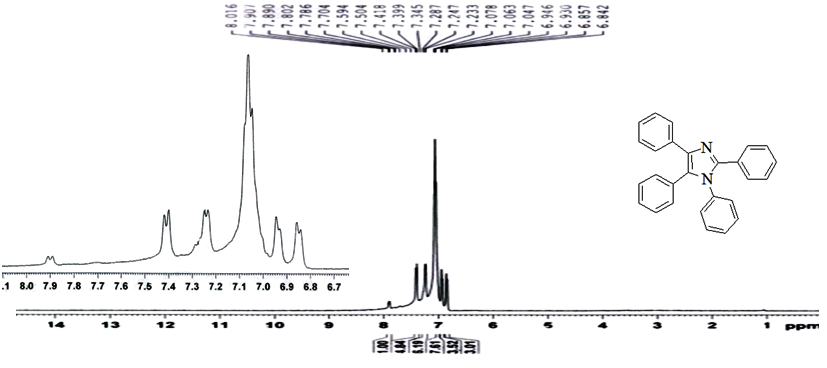


**The ^1^H NMR spectrum 1,4,5-Triphenyl-imidazole**
